# Supplementary material for: A 3D printed plant model for accurate and reliable 3D plant phenotyping
Source: Gigascience. 2024 Jun 20;13:giae035. doi: 10.1093/gigascience/giae035 (PMC11186670; doi:10.1093/gigascience/giae035)
Supplement: giae035_GIGA-D-24-00025_Original_Submission [file giae035_giga-d-24-00025_original_submission.pdf]

GigaScience

A 3D printed plant model for accurate sensor reconstruction and reliable 3D plant phenotyping

--Manuscript Draft--

**Manuscript Number:**	GIGA-D-24-00025
**Full Title:**	A 3D printed plant model for accurate sensor reconstruction and reliable 3D plant phenotyping
**Article Type:**	Research
**Funding Information:**	Bundesanstalt für Landwirtschaft und Ernährung (28DK108C20) — Prof. Dr. Anne-Katrin Mahlein<br>Bundesanstalt für Landwirtschaft und Ernährung (28DE104A18) — Prof. Dr. Anne-Katrin Mahlein<br>Deutsche Forschungsgemeinschaft (EXC 2070 – 390732324) — Not applicable
**Abstract:**	Background: This study addresses the importance of precise referencing in 3D plant phenotyping, which is crucial for advancing plant breeding and improving crop production. Traditionally, reference data in plant phenotyping relies on invasive methods. Recent advancements in 3D sensing technologies offer the possibility to collect parameters that cannot be referenced by manual measurements. This work focuses on evaluating a 3D printed sugar beet plant model as a referencing tool.<br>Results: Fused deposition modeling has turned out to be a suitable 3D printing technique for creating reference objects in 3D plant phenotyping. Production deviations of the created reference model were in a low and acceptable range. We were able to achieve deviations ranging from –10 mm to +5 mm. In parallel, we demonstrated a high dimensional stability of the reference model, reaching only ±4 mm deformation over the course of one year. Detailed print files, assembly descriptions, and benchmark parameters are provided, facilitating replication and benefiting the research community.<br>Conclusion: Consumer-grade 3D printing was utilized to create a stable and reproducible 3D reference model of a sugar beet plant, addressing challenges in referencing morphological parameters in 3D plant phenotyping. The reference model is applicable in three demonstrated use cases: evaluating and comparing 3D sensor systems, investigating the potential accuracy of parameter extraction algorithms, and continuously monitoring these algorithms in practical experiments in greenhouse and field experiments. Using this approach, it is possible to monitor the extraction of non-verifiable parameter and create reference data.<br>The process serves as a model for developing reference models for other agricultural crops.
**Corresponding Author:**	Jonas Bömer, M.Sc.<br>Institute of Sugar Beet Research<br>Göttingen, GERMANY
**Corresponding Author Secondary Information:**	
**Corresponding Author's Institution:**	Institute of Sugar Beet Research
**Corresponding Author's Secondary Institution:**	
**First Author:**	Jonas Bömer
**First Author Secondary Information:**	

Powered by Editorial Manager® and ProduXion Manager® from Aries Systems Corporation

Order of Authors:	Jonas Bömer<br>Felix Esser<br>Elias Ariel Marks<br>Radu Alexandru Rosu<br>Sven Behnke<br>Lasse Klingbeil<br>Heiner Kuhlmann<br>Cyrill Stachniss<br>Anne-Katrin Mahlein<br>Stefan Paulus
Order of Authors Secondary Information:	
Additional Information:	
Question	Response
Are you submitting this manuscript to a special series or article collection?	No
**Experimental design and statistics**<br><br>Full details of the experimental design and statistical methods used should be given in the Methods section, as detailed in our Minimum Standards Reporting Checklist. Information essential to interpreting the data presented should be made available in the figure legends.<br><br>Have you included all the information requested in your manuscript?	Yes
**Resources**<br><br>A description of all resources used, including antibodies, cell lines, animals and software tools, with enough information to allow them to be uniquely identified, should be included in the Methods section. Authors are strongly encouraged to cite Research Resource Identifiers (RRIDs) for antibodies, model organisms and tools, where possible.<br><br>Have you included the information requested as detailed in our Minimum Standards Reporting Checklist?	Yes

Powered by Editorial Manager® and ProduXion Manager® from Aries Systems Corporation

**Availability of data and materials**<br><br>All datasets and code on which the conclusions of the paper rely must be either included in your submission or deposited in publicly available repositories (where available and ethically appropriate), referencing such data using a unique identifier in the references and in the “Availability of Data and Materials” section of your manuscript.<br><br>Have you have met the above requirement as detailed in our Minimum Standards Reporting Checklist?	No
If not, please give reasons for any omissions below.<br><br>as follow-up to "**Availability of data and materials**<br><br>All datasets and code on which the conclusions of the paper rely must be either included in your submission or deposited in publicly available repositories (where available and ethically appropriate), referencing such data using a unique identifier in the references and in the “Availability of Data and Materials” section of your manuscript.<br><br>Have you have met the above requirement as detailed in our Minimum Standards Reporting Checklist?<br><br>"	Data will be uploaded upon acceptance for reviewing and be accessible by the reviewers.

manuscript tex

Click here to access/download;Manuscript;paper_v1.tex

OXFORD (GIGA)^n SCIENCE

GigaScience, 2023, 1–20

doi: xx.xxxx/xxxx
Manuscript in Preparation
Paper

PAPER

A 3D printed plant model for accurate sensor reconstruction and reliable 3D plant phenotyping

Jonas Bömer[1,*], Felix Esser[2], Elias Marks[3], Radu Alexandru Rosu[4], Sven Behnke[4], Lasse Klingbeil[2], Heiner Kuhlmann[2], Cyrill Stachniss[3], Anne-Katrin Mahlein[1] and Stefan Paulus[1]

[1]Institute of Sugar Beet Research (IfZ), Holtenser Landstraße 77, 37079 Göttingen, Germany and [2]Institute for Geodesy and Geoinformation (IGG), University of Bonn, Nussallee 17, 53115 Bonn, Germany and [3]Institute of Photogrammetry (IPB), University of Bonn, Nussallee 15, 53115 Bonn, Germany and [4]Institute for Computer Science VI - Autonomous Intelligent Systems (AIS), University of Bonn, Friedrich-Hirzebruch-Allee 8, 53115 Bonn, Germany

[*]Correspondence author. Jonas Bömer. Institute of Sugar Beet Research (IfZ), Holtenser Landstraße 77, 37079 Göttingen, Germany. E-mail: boemer@ifz-goettingen.de

Abstract

Background: This study addresses the importance of precise referencing in 3D plant phenotyping, which is crucial for advancing plant breeding and improving crop production. Traditionally, reference data in plant phenotyping relies on invasive methods. Recent advancements in 3D sensing technologies offer the possibility to collect parameters that cannot be referenced by manual measurements. This work focuses on evaluating a 3D printed sugar beet plant model as a referencing tool.
Results: Fused deposition modeling has turned out to be a suitable 3D printing technique for creating reference objects in 3D plant phenotyping. Production deviations of the created reference model were in a low and acceptable range. We were able to achieve deviations ranging from −10 mm to +5 mm. In parallel, we demonstrated a high dimensional stability of the reference model, reaching only ±4 mm deformation over the course of one year. Detailed print files, assembly descriptions, and benchmark parameters are provided, facilitating replication and benefiting the research community.
Conclusion: Consumer-grade 3D printing was utilized to create a stable and reproducible 3D reference model of a sugar beet plant, addressing challenges in referencing morphological parameters in 3D plant phenotyping. The reference model is applicable in three demonstrated use cases: evaluating and comparing 3D sensor systems, investigating the potential accuracy of parameter extraction algorithms, and continuously monitoring these algorithms in practical experiments in greenhouse and field experiments. Using this approach, it is possible to monitor the extraction of non-verifiable parameter and create reference data. The process serves as a model for developing reference models for other agricultural crops.

Key words: 3D plant phenotyping, 3D printing, reference

Background

Adapting agricultural crop production to increasingly challenging environmental conditions is critical for enhancing production levels and ensuring production security. Farmers face challenging growing conditions caused by various biotic and abiotic environmental factors. Cultivation of an appropriate crop variety is a successful approach to maintain stable yields and handle climatic changes [1]. However, plant breeding for novel crop varieties is a time-consuming process that requires a lot of labor-intensive and reliable information about the interaction of a crop genotype with its environment. The process of accessing this information in form of geometric or physiological properties of the plant is called plant phenotyping. Traditionally, the acquisition of plant characteris-

Compiled on: January 25, 2024.
Draft manuscript prepared by the author.

tics is associated with invasive and destructive methods, which is still the basis of modern plant breeding [2, 3]. Nevertheless, the development and now widespread availability of non-invasive measurement technologies has led to a new era in the phenotyping of plants [4, 5].

In the past decades, three-dimensional (3D) plant phenotyping was utilized for assessing geometric properties of plants. Advances in passive and active optical sensors, coupled with 3D reconstruction algorithms, provide the basis for precise high throughput and high resolution 3D analysis of above-ground plant structures, advancing 3D shoot phenotyping research [6, 7]. This research topic aims to access the intricate 3D structure of plants by measuring various morphological traits and gather data about a crops growth status, ultimately resulting in an enhanced comprehension of plant growth. Additionally, information about the 3D structure of a plant is essential for crop modeling [2] and can even be used to correct other optical sensor data [2, 8]. Combining 3D data acquisition with advanced analysis techniques enables measurement of diverse morphological growth parameters across various scales, ranging from the canopy to single plant and organ level [2, 9].

Based on their research, Scholz et al. [10] concluded that an automatic morphological parameter assessment using 3D models can fulfill breeders' needs for accurate phenotypic data. 3D sensor technology has advanced to the extent that close-range, high-resolution scanning with millimeter accuracy and precision is becoming increasingly affordable. A digital representation of a plant's structure in the form of a 3D model (point cloud or mesh), can be used to digitize morphological measurements down to the scale of individual organs [11, 12]. Additionally, digital representations offer the potential to develop new 3D characteristics of plants that cannot be captured by humans like plant volume or surface area, establishing them as important traits for plant breeding [13]. However, this potential has not been extensively explored. Extracting 3D characteristics of plants in high throughput and under field or greenhouse conditions presents several challenges regarding data acquisition with different sensor systems, as well as processing and analysis of the data [14]. Ultimately, all sensor types face the problem of validating their measurements.

Validation in high resolution 3D scanning requires reference data to produce accurate and reliable results. In 3D plant phenotyping, researchers deal with complex structured, small-scale objects. Many morphological features like plant height or leaf length can be retrieved from a 3D plant model using custom or commercially available analysis software like CloudCompare [15]. However, evaluating these feature extractions can be challenging. The validation of many parameters still rely on manual measurements or visual scoring by human experts, which is labor-intensive and has limited precision. The accuracy of visual scoring is often questioned, for example in disease assessment. Nutter et al. [16] documented the impact of human raters on the final disease score. Scholz et al. [10] used expert scoring as a reference for 3D assessment of morphological parameters. They concluded than human scoring is prone to errors and identified the quantification of small parameter variations as one of the greatest weaknesses of human scoring. Moreover, manual reference measurements, have limitations in properly representing the plant's 3D structure. Modern sensor technology and advanced parameter extraction algorithms pose a challenge to the use of manual measurements and visual scoring as reference data.

Additionally, we have identified three general categories of 3D parameters for plants. The first category includes parameters that can be evaluated by creating reference data through manual measurements. This is limited to single, easy-to-derive parameters, such as plant height or leaf length. Determining precise data for these parameters is relatively straightforward but time-consuming and often invasive [2, 17], which precludes the generation of time series data. Goldbach et al. [17] performed 3D measurements of leaf length and width and stem length and created reference data using invasive two dimensional (2D) scans from a flatbed scanner. The authors report that physical and computational bias affect their reference measurements due to the 3D nature of the measured objects. They also identify the noise of the reference data as the limiting factor of the accuracy of their 3D measurements. Nguyen et al. [18] used the same referencing method as Goldbach et al. [17] by utilizing 2D scans for leaf length, width, perimeter and area to determine the reconstruction accuracy of a developed 3D scanning system for plant phenotyping. They used a conventional plastic houseplant to validate their measurements. However, in order to access the 2D reference data, the plastic plant had to be cut, rendering it useless and providing no real advantage over using a biological plant.

Secondly, we refer to parameters that are typically accessed through visual scoring because they cannot be measured manually or only with great difficulty. This process is time-consuming and lacks high accuracy [16, 10]. An example for this category is the scoring of leaf attitude, which describes the overall leaf angle of a plant. This requires a considerable amount of work and often lacks the necessary precision. For instance, trained experts have scored the leaf angle either absolutely [10] or relatively [19]. However, the leaf angle can also be measured using either a protractor [20, 21] or a digital inclinometer [22], but this process is even more time-consuming.

The third category includes parameters that are not measurable by humans and can only be retrieved through the combination of advanced optical sensor techniques and computer algorithms. Possible examples of fine morphological and novel parameters that cannot be captured by human workers include the convex hull or the shadow cast of a plant. Computerized data acquisition and analysis enable the recording of parameters of all categories while being non-invasive, comprehensible, and resource-efficient. It is essential to monitor and reference the extraction of parameters of all categories to provide a reliable data source for future plant breeding.

Biskup et al. [23] made initial approaches to create a functional 3D reference shoot model. They used soybean leaves, fixed them to a flat board, and varied the angle of the board relative to a stereo camera setup to determine the accuracy of the setup for leaf angle determination. Similarly, Dandrifosse et al. [24] used plant leaves of known size, and fixed them to a flat board to determine the accuracy and precision of leaf area measurements for different leaf angles. Müller-Linow et al. [25] created a more comprehensive 3D model of a plant. They used a 3D plant model made of plywood with eight adjustable flat leaves to evaluate the leaf angle estimation of a custom stereo camera system with downstream data analysis and reported a good agreement between the values of the artificial plant model and the measured values. However, both approaches presented here only deal with the evaluation of a single morphological feature. After all, an artificial plant model can be used for referencing various morphological parameters. Under these circumstances, the need for a new reference method becomes apparent. Topp et al. [26] used 3D printing to manufacture a reference model of a simplified root system and used it to evaluate automatically extracted parameters. Since the 3D printed reference model is based on a digital model of the root system, extracted parameters can be compared to software-based reference parameters. Therefore, they emphasized the significant role of 3D printing in 3D phenotyping.

To address the subject of referencing in 3D shoot phenotyping, we transfer the idea of Topp et al. [26] of 3D printing reference structures for phenotyping to above ground plant structures and propose a 3D model of a sugar beet plant (*Beta vulgaris*) that can be produced using additive manufacturing methods (Figure 1). The increasing popularity of 3D printing in recent years has opened up new possibilities for the scientific community to quickly and easily create parts and prototypes. Fused deposition modeling (FDM) was used to manufacture the 3D reference model due to its reliability and affordability. This technique is widely used in various disciplines and is known for its ease of handling and reproduction, while maintaining high precision and quality standards [27, 28]. FDM 3D printing is cost-effective and requires minimal finishing work. At

the same time, it has low user requirements, making it a cutting-edge technique for rapid prototyping and component production that is accessible to everyone.

The main contribution of this paper is an exploration into the potential of utilizing a 3D printed plant model as a precise referencing tool in 3D plant phenotyping. Therefore, we first analyze the deviations between the underlying computer model and the 3D printed reference model. Next, we integrate a reference model into our research activities in field and greenhouse experiments over the course of one year. The dimensional stability over time is assessed using high-precision laser scanning and downstream deformation analysis. In order to demonstrate the practical applications of the reference model and contribute to solving the referencing problem in 3D plant phenotyping, we present three use cases:

A) Classify the suitability of a 3D sensor for plant phenotyping by generating precision and occlusion scores

B) Evaluate the accuracy of a parameter extraction algorithm under laboratory conditions

C) Monitor the stability of an automatic parameter extraction of verifiable and non-verifiable parameters in practical applications

In addition to providing the 3D model of the reference plant and a detailed construction manual, we offer benchmark parameters for various morphological parameters on both plant and single leaf scales, which have been extracted automatically using software-based approaches and manually using traditional measuring techniques. This enables other scientists to use the 3D reference model for their research, refine the methods used, and apply the gained knowledge to create reference models for other important agricultural crops. All required data is available at (Will be provided upon acceptance of the work).

Material and Methods

3D Reference Model

Data basis

The 3D reference model is based on a real sugar beet plant (Vasco, SESVanderHave N.V., Tienen, Belgium), cultivated under greenhouse conditions. At the time of data collection, the plant was approximately at BBCH 19. The workflow for creating the reference model is visualized in Figure 2. A light detection and ranging (LiDAR) scanner (Faro Focus S70, Faro Technologies Inc., Lake Mary, USA) was used to create high precision 3D point clouds from twelve different viewing angles. Using multiple spherical registration targets, the single scans were registered into one occlusion-free 3D representation of the sugar beet plant using the software Faro Scene. Next, the point cloud was processed using outlier removal algorithms implemented in the python library Open3D (v0.13.0) [29]. The surface of the point cloud was reconstructed with the help of a ball-pivoting algorithm [30] and smoothed using moving least squares surface reconstruction [31] implemented in the open source software CloudCompare (v2.11.1) [15]. After this, the resulting triangle mesh was loaded in blender (v2.92) [32] for further manual editing.

Manual editing

To make the leaf blades stable and 3D printable, they were thickened to approximately three millimeters using a solidify modifier. As sugar beet stems are comparatively delicate and fragile, it is very difficult to reproduce them in detail in a 3D printable model. It can be assumed that an accurate reproduced stem cannot withstand the stresses that occur, for example, due to transport of the printed model. For this reason, all stems were thickened using different sculpting tools available in blender, resulting in stronger stems that are more suitable to resist ordinary stresses. For the last design step, the bisect tool was used to split the reference model into 3D printable pieces. This step was mainly performed to control the layer orientation while 3D printing, since they strongly influence tensile force properties of 3D printed parts [33, 34]. Following this idea, the model was split into leaves and beet body, which was again split into three pieces. Other benefits from splitting the model into multiple parts are a lower printing failure rate due to multiple smaller prints and the possibility to print the reference model on 3D printers with a small build volume. The cut surface was designed in a v-shape in order to minimize deviations between the digital and the 3D printed model when assembling the individual parts later. After completing the manual editing steps, the individual parts were exported in .stl format.

3D printing

To produce the reference model via 3D printing, the individual model parts were loaded into the slicer software PrusaSlicer (v2.4.0-beta1) [35]. A slicer software takes a 3D object file and generates G-code instructions for the printer to fabricate the object layer by layer. A Prusa i3 MK3S+ printer (Prusa Research a.s., Prague, Czech Republic) was used together with the standard printer profile. Supports were activated on the build plate and the 0.15 mm quality profile was chosen and adjusted to our requirements (perimeters: 3, fill density: 50%, fill pattern: Gyroid, brim width: 5 mm, overhang threshold: 30°).

The orientation of the individual parts on the print bed is a critical part of the FDM production process, as the connection between the individual layers is weaker than the material itself [36]. For example, tensile forces should always be applied parallel to the layers, not orthogonal to them [37]. Applied to the reference model, this means that the stems should be oriented parallel to the print bed, as shown in Figure 2. In this way, tensile forces are optimally absorbed by the layers in a parallel direction and bending forces are absorbed orthogonal to the layers. The increase in stability due to optimal positioning of the individual parts on the print bed is the main reason for dividing the model into individual parts.

The filament material was chosen considering the need to withstand the conditions in field and greenhouse trials and ordinary stresses during transport. Essential material attributes, both in a general sense and for ensuring durability, encompass good mechanical properties alongside high tolerances for moderate temperatures, ultraviolet (UV) light, and humidity. Moreover, the filament should be easy to print and processable by most consumer grade 3D printers. Considering this, we've chosen Polyethylene terephthalate glycol (PETG) as the printing filament. It has a high mechanical resilience, is resistant to UV light and water, and has has an acceptable tolerance towards temperature deformation [33, 38]. In contrast to widely used polylactic acid (PLA), PETG is not biodegradable and therefore more stable over time, while showing competitive strength, toughness and elongation properties [38]. Additionally, it is also more flexible and therefore less likely to break on impact [33, 37]. During printing with PETG, limited warping deformation and a low shrinkage ratio can be observed [39]. The model was printed out of Geeetech PETG Green (Shenzhen Getech Technology Co., Shenzhen City, China) using the Prusa PETG profile in PrusaSlicer. The total time needed for printing all model parts with the mentioned hardware and slicer settings was roughly 70 hours.

For assembly, the individual parts were first cleaned of their support material. The individual parts were then glued together with cyanoacrylate adhesive. Finally, a heat set insert with a diameter of five millimeter was melted into the base of the beet body, which allowed it to be easily mounted to a steel rod and positioned in any conducted experiment trials (see Figure 1).

3D printed Model Inspection

The 3D printed model was scanned immediately after production using a high precision laser triangulation system to create an accurate digital copy of the model for later analysis. The system includes a line laser scanner (Perceptron ScanWorks V5, Perceptron Inc., Plymouth, USA) mounted on a mobile measuring arm (Romer Infinite 2.0, Hexagon AB, Stockholm, Sweden) and has a sub-millimeter accuracy. It was used in multiple studies regarding different high accuracy 3D phenotyping approaches [40, 41, 42, 43, 44, 9]. The system's scanning and reconstruction accuracy underwent evaluation and was found to be of excellent quality [40]. We've used anti-reflection spray (AESUB orange, Scanningspray Vertriebs GmbH, Recklinghausen, Germany) to further improve the quality of the resulting point cloud. The generated point cloud allows for a precise description of the deviations between the computer model of the reference model and the 3D printed model due to the production process. Scans performed later allowed for the monitoring of dimensional stability over a longer period of time.

Registration

To compare the point clouds, their respective coordinate systems had to be aligned and registered to minimise systematic errors. To achieve this, the 3D scans of the printed model were first manually aligned with the digital model as accurately as possible using CloudCompare. As there were no 3D correspondence points available, a point-to-plane iterative closest point algorithm (ICP) [45] was employed as a final registration technique. Only the beet body and the petiole base of the point clouds were utilized for the final registration to prevent the ICP algorithm from averaging deformations of the leaf apparatus, thus biasing the actual deviations. The region around the beet body is considered as robust to deformation while still offering distinct points and surfaces for precise registration.

3D Point Cloud Comparison

The multi scale model to model cloud comparison (M3C2) distance [46] was computed to analyse the production deviations and structural deformation over time. The M3C2 technique does not require fixed point correspondences and, unlike direct cloud-to-cloud comparison methods, is less sensitive to outliers and the quality of the point clouds themselves. It retains local structural features and does not require meshing of the point cloud. The calculation involves several steps. Initially, the surface normals are estimated and oriented. Subsequently, the mean surface change in the normal direction determines the distance between the two point clouds. For a comprehensive explanation of the algorithm, we refer to [46]. The calculation was performed using an implementation of the algorithm in the python library py4dgeo (v0.5.0) [47]. To finally determine the production deviations, the M3C2 distance between the high-resolution 3D scan immediately after the production of the printed model and the computer model was determined. Changes in deformation over time were assessed by measuring the distance between the initial 3D scan of the reference model after production and one of the two further 3D scans taken after 143 days and 361 days. During this period, the reference model was subjected to extensive testing in both greenhouse and field environments and underwent significant mechanical, temperature, and UV light related stress. Therefore, the later scan accurately portrays realistic stresses caused by various measurement applications and long-term use of the model.

Morphological Benchmark Parameters

To effectively utilize the reference model in practical applications, it is necessary to precisely collect the model's morphological parameters in a well defined and comprehensible way. The task is to gather clear benchmark parameters, which can be used to assess various sensors and algorithms. Two approaches were employed: an automatic software-based approach, which offers high precision and repeatability, and a manual approach, which represents conventional, manual measurement techniques. Since parameters like convex hull or projected leaf area cannot be measured manually, the software-based approach includes more parameters.

Automated extraction

The automatic parameters were extracted from the initial high-accuracy 3D scan of the printed model, taken immediately after production. We've used Python (v3.10) [48] for an automated parameter extraction, utilizing the libraries Open3D, NumPy (v1.24.4) [49], Potpourri3D (v0.0.8) [50], Alphashape (v1.3.1) [51] and Descartes (v1.1.0) [52] besides the python built-in modules. The following is intended as a reproducible description of all automatically extracted parameters.

On a single-plant scale, the model's HEIGHT was determined by calculating the difference between the highest and the lowest points of the point cloud. To obtain the WIDTH, the point cloud was first projected onto the xy-plane. Afterwards, the largest euclidean distance of the point cloud was determined. The calculation of the CONVEX HULL VOLUME and the CONVEX HULL SURFACE AREA of the model was performed by utilizing the Qhull [53] algorithm. To determine the PROJECTED LEAF AREA, a 2D mesh was generated by projecting the point cloud onto the xy-plane. Subsequently, the 2D surface area was calculated. The LEAF AREA of the model consists of the surface area of its individual leaves, explained in detail below.

On single-leaf scale, leaf length (petiole length + leaf blade length), leaf blade length, leaf blade width and leaf area parameters were assessed according to the European Union measurement guidelines for variety approval [19]. For this purpose, the point cloud of the model was first manually divided into individual leaves by using CloudCompare. The leaves were cropped as close to the beet body as possible. For a comprehensible measurement, it is essential to determine the coordinates of three specific points: petiole base, leaf blade base and leaf blade tip. To better access these points, the longitudinal axes of all leaves were manually aligned with the global x-axis and the transverse axis orthogonal to the global z-axis. The petiole base was determined by identifying the point with the lowest x-axis value, thanks to the previous transformation. Utilizing the heat method for distance computation [54], the leaf blade tip was determined as the point with the greatest distance over the surface of the point cloud to the petiole base point. The calculated distance represents the LEAF LENGTH.

To access the leaf base point in sugar beet, a universal definition that can be applied to all developmental stages of a leaf must first be established, as to our knowledge there is none. We suggest defining this spot based on a rapidly increasing leaf width. For this purpose, the width is measured starting at the petiole base and moving along the longitudinal axis towards the leaf tip in segments of 1.0 mm by fitting a polynomial curve to the top surface of the point cloud of a segment. Across all measured segments, the mean width is continuously calculated by averaging the mean of the central 50% of all measurements. If the current measured width of a segment exceeds the average width by a certain factor, the midpoint of the leaf base is located within the current segment. We recommend a factor value of 2.5, since it achieved the highest level of correlation with manual measurements of sugar beet leaves in 2D measurements.

After determination of the leaf blade base, the LEAF BLADE LENGTH was calculated as stated for the leaf length. The width of the leaf blade was measured orthogonally to the longitudinal axis of the leaf blade in segments of 1.0 mm using the heat method for distance computation [54]. The greatest width of all segments was recorded as the LEAF BLADE WIDTH. To measure LEAF AREA, the leaf blade was detached from the petiole at the leaf base orthogonally to the longitudinal axis and the point cloud of the leaf blade was meshed using a ball-pivoting algorithm [30]. The sum of all triangles represents the leaf area. The LEAF INCLINATION ANGLE

was measured for each leaf blade as the angle between the vertical and a line joining the leaf blade basis and the leaf blade tip to allow for angles <90°.

Manual extraction

In addition to automatically extracting morphological reference parameters, conventional measurements were also taken for parameters that allowed manual extraction using the 3D printed reference model. For single plant parameters, a folding rule was used to measure HEIGHT and WIDTH, while the NUMBER OF LEAVES was counted manually. For the purposes of quantifying SINGLE LEAF MORPHOLOGICAL PARAMETERS, the use of a ruler alone is insufficient as it cannot accurately portray 3D structures. To overcome this limitation, a narrow strip of tape was affixed to the reference model to connect two specific points of interest within its 3D structure. Subsequently, this tape was adhered to a flat sheet of paper where it could then be measured using a ruler. The LEAF INCLINATION ANGLE was measured using a digital inclinometer. The designated measurement points and methods for manual and automatic measurements remain consistent.

Use Cases in Plant Phenotyping

Evaluation of 3D sensors

The high accuracy and stability of the reference model enables the evaluation of sensor systems used for 3D phenotyping in the field in terms of accuracy and completeness of the plants reconstruction created with those systems. In this work, we evaluate the 3D reconstructions of a robotic field platform as a use case for the reference model as it is described by Esser et al. [55]. The robot is equipped with a multi-camera and a laser-line-scanner-based phenotyping system consisting of 20 DSLM-cameras and two laser line scanners. The multi-camera system's 3D reconstruction is performed using the PermutoSDF method [56], while the laser-based system uses position information retrieved from global navigation satellite system (GNSS) and an inertial measurement unit (IMU) to reference the position of every measurement. For multi-temporal phenotyping capabilities, the robot is equipped with a georeferencing system including two GNSS antennas and a real time kinematic (RTK) GNSS receiver. Figure 3 shows the field robot and the mounted sensors. For a detailed description of the sensor properties, their configuration, and the methods used for plant reconstruction we refer to [55].

To evaluate the reconstructions of the camera and the laser phenotyping system, we first generated a reference scan of the printed plant in the lab using the laser line scanning system utilized for the 3D printed model inspection. Afterwards, the model was scanned with the camera and laser phenotyping system of the robotic field platform to generate 3D point clouds for both systems. For evaluation, we were interested in the accuracy and completeness of the point clouds. The M3C2 point cloud distance metric to the reference scan was used to evaluate the accuracy. To value the completeness of the 3D model, the reference scan and the reconstructions of the robotic platform were spatially subsampled to a point distance of 5 mm using a voxel grid filter. Afterwards, the differences in the number of points to the reference were determined, valuing the completeness of the sensor systems' plant reconstruction.

Evaluation of parameter extraction algorithms

Another use case for the reference model is the evaluation of automatic approaches for plant and leaf parameter estimation, as we provide highly precise reference values for the most common parameters on plant and single leaf basis. To eliminate possible influences of the utilized 3D sensor, the approaches can be tested on the digital 3D model of the reference model. Following this idea, we present a performance evaluation of an approach to autonomously measure the leaf length, leaf blade length, and the leaf blade width introduced by Marks et al. [11].

The approach is based on fitting an a priori leaf model to the 3D point cloud of the reference model. The a priori model is defined as a triangular mesh and represents the standard shape of a sugar beet leaf. This model was then deformed onto the point cloud in the fitting process, in order to obtain a triangular mesh that represents the specific leaf. We then extracted the parameters based on the deformed leaf model. For a more detailed explanation of the algorithm we refer to [11].

Continuous parameter extraction monitoring

The reference model can be used to continuously monitor the stability of an automatic parameter extraction in the combined system of sensors and algorithms. Therefore, it is necessary to repeatedly collect morphological parameters of the reference model in various test scenarios and environmental conditions. The approach can evaluate both verifiable and non-verifiable morphological parameters and reduce the need for labor-intensive and destructive manual reference measurements.

To demonstrate this use case for the verifiable parameters plant height and width and the non-verifiable parameter volume of the convex hull, the reference model was integrated into our standard process of 3D data acquisition using a LiDAR scanner in several greenhouse and field trials cultivating sugar beet (Figure 1). The reference model was positioned at different angles and locations relative to the sensor to maximize the variability of structural influences like distance or angle of incidence as described by Medic et al. [57]. After generating a 3D point cloud from the individual scans of the LiDAR sensor, the reference plant was extracted manually. The parameter extraction algorithms analyzed the point cloud of the reference model and extracted the height, the width and the volume of the convex hull of the reference model. The distribution of these parameters was examined and compared with the specified benchmark parameters.

Results

3D printed Model Evaluation

Production deviations

Even though recent 3D printers achieve high dimensional accuracy in their prints, there may be variations between the computer model and the final 3D printed reference model due to the assembly of multiple smaller printed parts. In order to evaluate dimensional differences caused by production, the M3C2 distance and the frequency of deviations were calculated (see Figure 5). Positive and negative deviations in the direction of the surface normals are observable. Some leaves exhibit uniform deviation while others show both positive and negative deviations on the same surface, indicating torsional distortion of the leaves. Deviation values range from approximately −10 mm to +5 mm. The average deviation measures −2.5 mm.

Dimensional stability

To assess the 3D printed reference model's dimensional stability over time, two high-precision 3D measurements were conducted (see Figure 6). The initial scan was performed immediately after production, while the second measurement was taken after 143 days (Figure 6 A). The reference model underwent only slight dimensional deformation ranging from −2 mm to +4 mm during the examined period. The average deformation was +0.2 mm. Most surfaces experienced deformation in the range of 0 mm to −1 mm. However, it is apparent that the deformations are specific to the individual leaves. It can be observed that the more extreme deformation values can be assigned to individual leaves that are either lowering or raising. These results are also evident in the reference model's second scan after 361 days (see Figure 6 B). The dimen-

Table 1. Morphological parameters of the 3D reference model at plant scale.

Parameter	Automated	Manual
Height [cm]	27.7	28.0
Width [cm]	45.1	45.5
Convex hull [cm^2]	3635	-
Convex hull [cm^3]	16107	-
Leaf area [cm^2]	809	-
Projected leaf area [cm^2]	522	-
Leaf count	-	12

sional deformations found in this case were between −4 mm and +4 mm and thus have only a slightly larger range compared to the previous scan. The average deformation was −0.1 mm. In addition, it is again evident that the leaves of the reference model do not deform uniformly. Overall, the average M3C2 distance values suggest a slight trend toward negative deformation in the direction of gravity.

Morphological Benchmark Parameters

Morphological reference parameters were extracted at both the single plant and single leaf scales using automated software-based and manual methods outlined in the section above. Results for the single plant parameters are presented in Table 1. There are only slight differences observed between the digital and manual extraction of a parameter. Extracted parameters for individual leaves are presented in Table 2, where the differences between the two extraction techniques are more noticeable. The average deviation between the two methods is 1.2 cm for the leaf length, 1.2 cm for the blade length, 0.6 cm for the blade width and 3.8° for the leaf angle.

Use Cases in Plant Phenotyping

Evaluation of 3D sensors

Figure 7 shows the M3C2 distances of the sensors of the robotic phenotyping platform to the reference model for one example leaf. The point clouds of the camera and laser sensor systems are registered to the reference scan using the ICP algorithm. The M3C2 histograms for both sensor systems are showing distances in the range of ±2 mm (Figure 7). The shapes of the histograms are similar to a normal distribution, but systematics can be recognized if looking at the point clouds colored according to the M3C2 distances in the upper part of the figure. Since Figure 7 just shows the results for one example leaf, the standard deviations of the M3C2 distances to the reference model were computed and are summarized in Table 3. Note that results for just ten leaves are shown here since occlusions of leaves 11 and 12 result in very incomplete reconstructions for both the camera and laser scanning system. The mean standard deviations over all ten leaves for the laser system and camera systems are valued at 0.28 mm (laser) and 0.45 mm (camera) at a maximum of 0.77 mm (laser) and 1.18 mm (camera).

These results show that both systems deliver reconstructions with an accuracy in the order of millimeters in most cases. Table 3 also shows the number of points after subsampling the point clouds of the reference, laser, and camera reconstruction. The difference in the number of points and their percentage to the reference is used to value the completeness of the reconstructions (occlusion factor). For the laser point cloud, the mean point difference over all ten leaves is 9.23% at a maximum of 15.1% for leaf 7. The results for the camera system show more complete reconstructions at a mean point difference of 5.19% at a maximum of 13.8% for leaf 10.

Evaluation of parameter extraction algorithms

We used the point cloud of the reference plant collected by a high precision laser scanner to validate the template fitting approach presented in [11]. The results are reported in Table 4. The approach performed particularly well for leaf length and blade width estimation, where the mean errors are 4.1% and 4.2% respectively. There appears to be a slight trend towards higher algorithmic errors for smaller leaves located in the center of the plant. The blade length estimation with a mean error of 10.9% was more error prone than the estimation of the other two parameters. Figure 4 shows the fitted leaf model for two exemplary leaves.

Continuous parameter extraction monitoring

To assess the reliability of an automatic parameter extraction, we integrated the reference model into our standard 3D data acquisition procedure. Thereafter, algorithms were employed to automatically gather morphological characteristics. Table 5 exhibits the measurements for the height, width, and volume of the convex hull for nine measurement dates. Based on the data collected, there is no noticeable difference in the measured values between greenhouse and field trials. The measured values for the parameters height and width have a maximum deviation of 1.4% from the reference value. There is an average deviation of 0.2 mm or 0.8% observed for the height, and 0.2 mm or 0.5% for the width. The volume of the convex hull shows a maximum deviation of 8.2% from the reference value. On average, the measured volume of the convex hull deviates from the reference by 471.0 cm^3 or 2.9%. It can be observed that, with the exception of one measurement, the evaluated parameters always deviate downwards and are therefore lower than the reference parameters.

Table 2. Morphological parameters of the 3D reference model at leaf scale.

Leaf	Automated					Manual			
	Leaf length [cm]	Blade length [cm]	Blade width [cm]	Leaf angle [°]	Leaf area [cm^2]	Leaf length [cm]	Blade length [cm]	Blade width [cm]	Leaf angle [°]
01	25.4	14.6	9.1	108.7	100.9	27.6	15.6	9.3	104.0
02	26.4	12.0	8.2	65.7	69.6	25.9	14.2	8.4	63.0
03	27.8	14.8	9.2	87.0	92.9	26.4	15.5	10.1	85.0
04	29.0	13.5	8.3	42.3	80.7	29.1	14.4	9.1	42.0
05	27.5	14.3	8.7	21.9	90.7	31.9	16.7	10.5	20.0
06	32.0	16.6	10.2	47.8	121.2	28.4	14.4	9.4	49.0
07	29.1	14.4	9.3	40.4	90.7	27.6	16.1	9.1	41.0
08	23.5	11.6	7.2	28.8	60.6	22.3	11.1	7.0	30.0
09	22.4	10.4	6.8	26.7	51.0	23.4	11.6	7.8	18.0
10	17.9	8.9	4.8	19.8	30.5	18.0	9.3	4.9	12.0
11	17.9	7.4	2.3	17.4	10.0	17.9	8.0	2.4	10.0
12	14.0	8.1	2.3	10.8	10.4	13.1	7.5	1.9	4.0

Table 3. M3C2 standard deviations and number of points per leaf after subsampling for the robot's laser and camera systems to reference scan. The percentage values indicate the deviation from the reference values.

Leaf	σM3C2 [mm]		Number of points		
	Laser	Camera	Laser	Camera	Ref
01	0.24	1.18	363 (-7.6%)	382 (-2.8%)	393
02	0.20	0.57	267 (-14.9%)	306 (-2.5%)	314
03	0.20	0.48	399 (-6.6%)	387 (-9.4%)	427
04	0.22	0.47	297 (-8.9%)	340 (+4.3%)	326
05	0.34	0.28	322 (-14.6%)	366 (-2.9%)	377
06	0.32	0.32	354 (-4.1%)	355 (-3.8%)	369
07	0.77	0.29	321 (-15.1%)	362 (-4.2%)	378
08	0.12	0.20	204 (-2.9%)	205 (-2.4%)	210
09	0.37	0.32	226 (-7.0%)	229 (-5.8%)	243
10	0.07	0.36	110 (-10.6%)	106 (-13.8%)	123
mean	0.28	0.45	9.23%	5.19%	-

Table 4. Automatically estimated morphological parameters of the 3D reference model at leaf scale. The percentage values indicate the deviation from the automatically collected benchmark values.

Leaf	Leaf length [cm]	Blade length [cm]	Blade width [cm]
01	26.1 (+2.8%)	15.6 (+6.8%)	9.9 (+8.8%)
02	27.7 (+4.9%)	14.4 (+20.0%)	8.3 (+1.2%)
03	29.3 (+5.4%)	17.8 (+20.3%)	9.2 (+0.0%)
04	30.5 (+5.2%)	15.9 (+17.8%)	8.8 (+6.0%)
05	28.1 (+2.2%)	14.6 (+2.1%)	8.7 (+0.0%)
06	33.1 (+3.4%)	16.7 (+0.6%)	10.1 (-1.0%)
07	29.5 (+1.4%)	14.2 (-1.4%)	9.5 (+2.2%)
08	23.7 (+0.9%)	10.4 (-10.3%)	7.2 (+0.0%)
09	23.4 (+4.5%)	12.1 (+16.3%)	6.3 (-7.4%)
10	19.5 (+8.9%)	9.0 (+1.1%)	4.7 (-2.1%)
11	18.8 (+5.0%)	9.3 (+25.7%)	2.2 (-4.3%)
12	14.7 (+5.0%)	7.4 (-8.6%)	1.9 (-17.4%)
mean	4.1%	10.9%	4.2%

Table 5. Monitoring the parameter extraction from the reference model in greenhouse and field experiments at different time points. The percentage values indicate the deviation from the automatically collected benchmark values.

Time	Height [cm]	Width [cm]	Convex hull volume [cm^3]
01[a]	27.3 (-1.4%)	44.8 (-0.6%)	16091.3 (-0.1%)
02[a]	27.5 (-0.9%)	44.5 (-1.4%)	15705.8 (-2.5%)
03[a]	27.4 (-1.0%)	45.0 (-0.2%)	16029.8 (-0.5%)
04[a]	27.4 (-1.0%)	44.7 (-0.8%)	15690.3 (-2.6%)
05[a]	27.4 (-1.1%)	44.7 (-0.9%)	15561.6 (-3.4%)
06[b]	27.7 (±0.0%)	45.1 (±0.0%)	15610.0 (-3.1%)
07[b]	27.7 (±0.0%)	44.9 (-0.4%)	15473.0 (-3.9%)
08[b]	27.4 (-1.1%)	45.1 (±0.0%)	14791.0 (-8.2%)
09[b]	27.8 (+0.4%)	45.2 (+0.2%)	15774.0 (-2.1%)
mean	0.2 (-0.8%)	0.2 (-0.5%)	471.0 (-2.9%)

a = greenhouse; b = field

Discussion

3D Reference Model

3D printing for referencing in 3D plant phenotyping

In recent years, the use of 3D printing has increased in different scientific domains, including plant science. Griffiths [58] analyzed the applications of 3D printing in plant science and found that in addition to the production of usable plant growth systems (71.4%) and phenotyping tools (14.3%), 3D printing is already being used for modeling and analysis validation (14.3%). However, so far this usage is limited to the rhizosphere. Liang et al. [59] utilized a 3D printed artificial model of a tree root cluster to investigate the response of vegetated slopes exposed to earthquake ground motion using geotechnical centrifuge modeling. Although this application differs significantly from ours, Topp et al. [26] employed 3D printing to fabricate a reference model of a simplified root system and used it to evaluate automatically extracted parameters like the number of roots or the convex hull volume. These were the first studies to employ 3D printing technology for validation purposes in plant phenotyping. They concluded that printing of plant parts can provide important reference data for 3D parameter analysis. However, the given examples are limited to laboratory use. Our reference model is designed for use in outdoor environments of field trials and is therefore exposed to challenging environmental factors like heat, UV light, and moisture that can alter the model's dimensions over time.

To validate our proposed plant model as a reference object, the first step was to analyze the production deviations between the computer model and the 3D printed reference model to determine the precision of our manufacturing process. As shown in Figure 5 we were able to achieve deviations ranging from −10 mm to +5 mm using FDM 3D printing technology. The deviation is believed to be primarily due to the assembly process rather than low reproduction accuracy of the single model parts. Large laminar deviations are not present, and modern 3D printers have demonstrated excellent reproduction accuracy [27, 28]. The v-shaped connection of the leaves requires manual vertical orientation, which may result in lowered or raised leaf surfaces. Possible improvements of this connection are discussed below. However, the deviations that were evaluated appear to be negligible for many morphological parameters, such as plant height, width, or the length of individual leaves. The morphological parameter that appears to be the most affected is supposedly the leaf angle.

To quantify possible dimensional stability problems, the reference model was examined 143 and 361 days after production. In between, the reference model was used in greenhouse and field experiments and subjected to intense external influences such as UV light, temperature and humidity, which are known to affect the properties of thermoplastic polymers such as PETG [60]. Nevertheless, the reference model shows only small dimensional deviations of ±4 mm over the course of nearly one year, with the absolute mean deviation being close to 0 mm (Figure 6). This demonstrates the stability and usability of our 3D printed reference model for the use in different environments. Two main effects of deformation are expected to take place. The first effect is internal to the leaf, meaning that it affects the shape or size of the leaf blade. The second effect affects the positioning of the leaf blade by altering its orientation or positioning through a bending of the petiole. Figure 6 shows that petiole deformation has the greatest effect on overall deviations, as evidenced by the more or less uniform coloration of individual leaves. Altogether, there is a slight tendency for the leaves to descend. However, this is not the case for all leaves of the reference model. To determine the service life of a 3D printed reference model, the dimensional stability should be analyzed furthermore. Possible solutions to improve the dimensional stability over time are discussed below.

The analysis demonstrates that consumer grade FDM 3D printers can accurately print even complex structured models of agricultural crops. The reference model is reproducible and fulfills necessary requirements for a use in plant phenotyping in terms of accuracy and dimensional stability. Therefore, extracting morphological parameters from 3D printed plant models for validation and referencing purposes is a reasonable approach.

Improving production precision and model persistence

Although the production of the reference model is straightforward, there are some ways to improve it to enhance ease of production and use, reduce production deviations (illustrated in Figure 5), and improve dimensional stability (analyzed in Figure 6). These improvements will likely enhance the precision and persistence of the reference model and can be considered when reproducing it. However, it is important to note that the improvements to be discussed are optional. The current setup is already sufficient with respect to the requirements in 3D phenotyping.

The design of the v-shaped connection between the beet body and the leaf stems is a major contributor to production deviations. This connection prevents false lateral alignment but can result in incorrect vertical positioning of the leaf, as demonstrated in Figure 5. An angled connector would resolve this issue and substantially decrease the deviation between the computer model and the 3D printed version of the reference model. Another approach for improving the connection could be to embed precast connectors during the printing process. Embedding objects during 3D printing is a technique used in various disciplines and has the potential to add new functionalities to the printed parts [61]. It may even be possible to embed quick connectors to allow for disassembly of the reference model. However, further research and development is needed to validate these reconstructions in terms of production deviations and temporal stability.

The choice of filament has a strong impact on production deviation and dimensional stability. Warping of printed parts during production due to cooling can cause deviations on a small to medium scale and is directly linked to the material properties of the filament used. PETG was chosen over acrylonitrile butadiene styrene (ABS) or polyamide (PA) due to its low tendency for warping and superior mechanical properties compared to PLA, while still being as easy to process as PLA. A recent advancement in 3D printing involves the utilization of fiber-reinforced filaments. These filaments consist of a thermoplastic polymer, such as PETG, as a matrix material and are strengthened with carbon, aramid, or glass fibers. The application of these engineered materials appears suitable due to their enhanced elasticity and ultimate tensile strength, as described by Kannan et al [62]. However, it is crucial to reassess production deviations and dimensional stability over time when using a new filament type, as the addition of fibers to PETG can alter characteristics such as the shrinkage ratio [63].

Additionally, the positioning of the leaves on the print bed is believed to affect print deviation and dimensional stability over time. Figure 2 shows that the leaves were printed as a whole in a lateral position, which may cause the stem, being the weakest part, to warp upwards or develop unilateral internal stresses that bend the stem over time. As a countermeasure, Figure 8 proposes an improved printing approach for a single leaf. The leaf is cut along its longitudinal axes and assembled after printing. This method minimizes warping due to the large contact surface of the stem with the print bed and generates counteracting internal stresses in the stem, leading to reduced deformation over time. Furthermore, this technique significantly reduces the need for support structures by eliminating overhanging parts, which minimizes both printing time and waste. In addition, when combined with a variable layer height, as shown in Figure 8, it has the potential to improve the surface texture of the printed objects.

The Gyroid infill pattern was chosen for its isotropic properties [36]. However, it is worth noting that PETG printed parts exhibit improved mechanical properties with increasing infill density [34]. According to Sepahi et al. [38], PETG printed parts reach their optimal tensile strength when printed with an infill pattern parallel to the direction of loading. Therefore, it may be more beneficial to print the single leaves with a dense infill pattern linear to the stem. This can be achieved by using a high number of perimeters. When fiber-reinforced filaments are combined with it, the reference model's resilience can be significantly enhanced.

Benchmarking parameters

To increase the applicability of the reference model, benchmark parameters have been collected both automatically and manually for a variety of use cases. Table 1 contains the extracted plant-based parameters, while leaf-based parameters are listed in Table 2.

The plant parameters show good accordance between the manual and the automated measurement method, while the deviations are considerably higher for leaf parameters. However, it is important to note that comparisons are made at a high level of precision, making manual measurements difficult to use. The observed differences are likely to be mainly due to the difficulty of maintaining consistent measurement points and rules when performing manual measurements, and are likely to be less affected by systematic measurement errors of one or the other approach. This situation highlights the challenge of comparing measurements obtained using manual and software-based methods. Determining the points of the petiole base and leaf blade base can be challenging, as previously noted. This applies to both manual and software-based measurements, as well as comparisons between different algorithms. To address this, benchmark parameters were collected in a clear and traceable manner.

A subsequent implementation of novel 3D morphological parameters for plant phenotyping applications is conceivable. Given the high point accuracy and density of the original 3D reference model, it is feasible to collect measurements for new parameters on an idealized database. Later, these measurements can be compared to real-world data, which may be biased by environmental influences, data collection procedures, or the use of a lower quality sensor for data acquisition.

Use Cases in Plant Phenotyping

Evaluation of 3D sensors

Based on the extracted benchmark parameters and the inclusion of novel morphological 3D parameters for plant phenotyping, the 3D reference model can be applied to numerous use cases. The first use case involves comparing different 3D sensors and their use for 3D phenotyping. This is a crucial topic as different sensor types use different physical approaches to acquire data, which can have an individual influence on the resulting data structure and its accuracy [14]. In addition, different sensor types require special positioning or data recording procedures for the plant, which affects the sensor's field of view and, consequently, the occlusion rate it can achieve.

Esser et al. [55] previously compared the used laser and camera sensor systems using a real plant, similar to our approach. However, their comparison was based on an unstable living object that is not available to other researchers. By using a printed 3D reference model, our comparison provides greater context and better reproducibility and enables other research facilities to compare their 3D sensor systems and track technological progress in this area. Table 3 displays the results of the sensor comparison conducted in this study. It is noteworthy that the laser system, while twice as accurate as the camera system, has a significantly higher occlusion rate. The camera system produces more complete reconstructions, which can be attributed to it's sensor configuration. Equipped with 20 cameras, it measures the plants from multiple angles, resulting in a more complete reconstruction compared to the laser scanning system which only observes from the right and left sides. However, none of the systems were able to reconstruct the two innermost leaves of the reference model to a usable degree. This demonstrates the potential of the reference model in identifying weak points in reconstruction, which can be addressed by the development of future 3D sensor systems and reconstruction algorithms.

The results shown here highlight the potential of the 3D reference model for the evaluation of 3D phenotyping systems by making a statement about the accuracy and completeness of their recon-

structions.

Evaluation of parameter extraction algorithms
Our second use case involves the use of the reference model to evaluate algorithms for extracting morphological parameters at the plant and leaf level. This is achieved by comparing the output of an algorithm with the automatically or manually extracted precise benchmark parameters.

Precise definition of the measurement parameters is crucial at the high level of accuracy enabled by modern 3D sensors. Scholz et al. [10] identified issues when comparing results from two measurement methods due to differing definitions used to record values. Golbach et al. [17] described the noise in (manual) reference data as the limiting factor for the accuracy of 3D measuring approaches. This limitation applies especially to measuring parameters such as leaf angle, which are typically difficult to access through manual measurements [20, 21, 22] or human scoring [10, 19]. The automatically morphological measurements conducted in this study are precisely defined and can be imitated. They serve as benchmark values obtained under optimized conditions. These values can be used to evaluate the performance of a tested algorithm intended for practical use with an optimal data basis.

To simulate this use case, a state-of-the-art algorithm by Marks et al. [11] for estimating single leaf morphological parameters of sugar beet was investigated. It was developed to estimate the total leaf length, leaf blade length, and leaf blade width under real field conditions and with respect to incomplete point clouds by detecting key points of sugar beet leaves. The results are presented in Table 4. The algorithm's performance on real-world data is reasonable for leaf length and leaf width, which are in good agreement with the benchmark values. Figure 4 displays two examples of the leaf model fitted to the point cloud of the reference model, demonstrating the approach's effectiveness. However, the estimation of the length of the leaf blade exhibits a higher mean error rate. This is likely due to the difficulty in detecting the joint between the petiole and the leaf blade, as well as the ambiguity in the definition of this point (see above).

This observation highlights the importance of defining key measurement points accurately. Another example of this is the consistently positive deviation in leaf length observed in the algorithm used (see Table 4). It can be concluded that the definition of the stem base should be investigated and optimized. In this use case, the ability to detect systematic errors in algorithms used to extract morphological parameters was demonstrated. Based on this, fine-tuning can be performed to match the recorded values with the provided benchmark parameters. Furthermore, this data can be used to compare different parameter extraction algorithms among each other.

Continuous parameter extraction monitoring
The third use case utilizes the 3D reference model as a stable reference object for automatic parameter extraction in scientific experiments. This approach offers two main benefits: evaluating the measurement system (interaction of sensor and algorithm) used for plant phenotyping under practical conditions and creating verification data for both verifiable and non-verifiable morphological parameters that lack necessary reference data.

The study demonstrates that the used measurement system performs well in various environments, allowing for high precision monitoring at millimeter scale of specific 3D parameters. The results also indicate that the measurement system is relatively insensitive to changes in distance and angle of incidence between the reference model and the sensor, as shown in Table 5. The results confirm the accuracy of the approach used without requiring intensive manual labor for reference measurements. They also indicate that it is possible to reference parameter extractions for 3D parameters that are not verifiable under normal circumstances, such as the volume of the convex hull. However, time point eight shows a significant decline in the measured value for the convex hull (Table 5). Simultaneously, the measurements for plant height and width show no unusual behavior. A visual inspection confirmed the integrity of the underlying point cloud. This leads to the conclusion, that higher dimensional parameters, such as the volume of the convex hull, are more prone to measuring errors than one-dimensional parameters like plant height or width. Therefore, high dimensional parameters generally exhibit greater parameter variation and tend to have more measurement outliers. With this in mind, the proposed 3D reference model provides the opportunity to monitor these types of parameter extractions.

Regarding the implementation of new 3D phenotyping parameters, this discovery allows the user to measure the stability and usefulness of novel parameters that can only be obtained through the combination of software-based data acquisition and subsequent data analysis. Therefore, only stable 3D parameters are useful for 3D phenotyping.

The proposed use case allows the user to easily evaluate whether the required tolerances in automatic parameter extraction are met or not. However, this workflow has a limitation in that it cannot automatically identify the source of errors, but rather simplifies the evaluation process. Measurement errors must be manually determined as either due to a defective 3D model by the 3D sensor (such as scale errors, high occlusion rates, or outliers) or caused by processing algorithms (such as faulty preprocessing, segmentation, or extraction errors).

Future Scenarios

As a first step for further research, the proposed improvements to the production process should be investigated. These improvements can contribute to better production accuracy and resilience, thus extending the use of the model. In addition, consideration should be given to complementing the reference model of a sugar beet with additional individual reference models at different growth stages to demonstrate a wider range of morphological characteristics and 3D architecture.

The use of 3D printing for creating reference objects in plant phenotyping is not restricted to sugar beet and the leaf apparatus. We see an opportunity to apply this concept to other cultivated crops, even if they have unique design and production requirements. Rosulate plants appear to have an advantage in recreating the leaf apparatus, while creating a 3D printable wheat plant model presents a challenge. However, the creation of a 3D reference model seems to be possible for juvenile growth stages of most agricultural crops. Additionally, the proposed workflow could be adapted to horticultural production to produce 3D printed fruits and vegetable references. This would be useful for tasks such as monitoring 3D shape completion, as performed by Magistri et al. [64] and Pan et al.[65], for use in robot automated greenhouses.

Conclusion

Consumer-grade FDM 3D printing enables to produce highly accurate and stable reference models for application in 3D plant phenotyping, contributing to solving the issue of referencing morphological parameter extractions. The proposed reference model is accurately reproducible and stable over a longer period, indicating that FDM 3D printing is a suitable production technique for the suggested applications. The introduced process of creating a 3D reference model for sugar beets can serve as an example for developing similar reference models for other widely-used arable or horticultural crops. Through the experiments conducted, it was determined that 3D reference models can serve a wide range of applications in 3D plant phenotyping. Besides representing a standardized approach for comparing 3D sensor systems based on their accuracy

and reconstruction completeness of plants, an evaluation of the precision of parameter extraction algorithms designed for high throughput phenotyping under ideal conditions was demonstrated using our reference model. Additionally, the reference model is used to monitor the extraction of morphological parameters under practical conditions. Therefore, it is possible to provide verification data for 3D morphological parameters, that can not be referenced using traditional referencing methods used in plant phenotyping. We provide files and a detailed description for reprinting the model, along with precise manual and automated benchmark parameters for plant and single leave parameters, enabling the research community to replicate and benefit from our research.

Data availability

Will be provided upon acceptance of the work using GigaDB.

Declarations

List of abbreviations

2D: two dimensional; 3D: three dimensional; ABS: acrylonitrile butadiene styrene; FDM: fused deposition modelling; GNSS: global navigation satellite system; ICP: iterative closest point; IMU: inertial measurement unit; LiDAR: light detection and ranging; M3C2: multi scale model to model cloud comparison; PETG: polyethylene terephthalate glycol; PLA: polylactic acid; PA: polyamide; RTK: real time kinematics; UV: ultraviolet

Ethical Approval (optional)

Not applicable

Consent for publication

Not applicable

Competing Interests

The authors declare that they have no competing interests.

Funding

The project is supported by funds of the Federal Ministry of Food and Agriculture (BMEL) based on a decision of the Parliament of the Federal Republic of Germany. The Federal Office for Agriculture and Food (BLE) provides coordinating support for artificial intelligence (AI) in agriculture as funding organisation, grant number 28DK108C20, and under the strategy for digitization in agriculture, grant number 28DE104A18. Additionally, this work has partially been funded by the Deutsche Forschungsgemeinschaft (DFG, German Research Foundation) under Germany's Excellence Strategy – EXC 2070 – 390732324.

Author's Contributions

Conceptualization: JB, SP
Methodology: JB, FE, EM
Investigation: JB, FE, EM, RAR
Visualization: JB, FE, EM
Funding Acquisition: AKM, SP, CS, LK, HK, SB
Project Administration: AKM
Supervision: SP, AKM, LK, CS, SB, HK
Writing – Original Draft: JB, FE, EM
Writing – Review & Editing: JB, FE, EM, RAR, SB, LK, HK, CS, AKM, SP

Acknowledgements

We would like to thank our colleagues from IfZ, IGG, and AIS for their contributions, inspiration, discussions, and support related to this research. We especially thank Saskia Flentje and Jonathan Eggers for their technical support. Furthermore, we would like to express our gratitude to the German Federal Plant Variety Office for allowing us to gather data in their field trials.

References

1. Korres NE, Norsworthy JK, Tehranchian P, Gitsopoulos TK, Loka DA, Oosterhuis DM, et al. Cultivars to face climate change effects on crops and weeds: a review. Agronomy for Sustainable Development 2016;36:1–22.
2. Fiorani F, Schurr U. Future scenarios for plant phenotyping. Annual review of plant biology 2013;64:267–291.
3. Pieruschka R, Schurr U. Plant Phenotyping: Past, Present, and Future. Plant Phenomics 2019;2019:7507131. https://spj.science.org/doi/abs/10.34133/2019/7507131.
4. Furbank RT, Tester M. Phenomics–technologies to relieve the phenotyping bottleneck. Trends in plant science 2011;16(12):635–644.
5. Watt M, Fiorani F, Usadel B, Rascher U, Muller O, Schurr U. Phenotyping: new windows into the plant for breeders. Annual review of plant biology 2020;71:689–712.
6. Paulus S. Measuring crops in 3D: using geometry for plant phenotyping. Plant methods 2019;15(1):1–13.
7. Harandi N, Vandenberghe B, Vankerschaver J, Depuydt S, Van Messem A. How to make sense of 3D representations for plant phenotyping: a compendium of processing and analysis techniques. Plant Methods 2023;19(1):1–46.
8. Behmann J, Mahlein AK, Paulus S, Dupuis J, Kuhlmann H, Oerke EC, et al. Generation and application of hyperspectral 3D plant models: methods and challenges. Machine Vision and Applications 2016;27:611–624.
9. Schunck D, Magistri F, Rosu RA, Cornelißen A, Chebrolu N, Paulus S, et al. Pheno4D: A spatio-temporal dataset of maize and tomato plant point clouds for phenotyping and advanced plant analysis. Plos one 2021;16(8):e0256340.
10. Scholz O, Uhrmann F, Wolff A, Pieger K, Penk D. Determination of detailed morphological features for phenotyping of sugar beet plants using 3D-stereoscopic data. ISPRS Annals of the Photogrammetry, Remote Sensing and Spatial Information Sciences 2019;4:161–166.
11. Marks E, Magistri F, Stachniss C. Precise 3D reconstruction of plants from UAV imagery combining bundle adjustment and template matching. In: IEEE International Conference on Robotics and Automation (ICRA); 2022. p. 2259–2265.
12. Wahabzada M, Paulus S, Kersting K, Mahlein AK. Automated interpretation of 3D laserscanned point clouds for plant organ segmentation. BMC bioinformatics 2015;16:1–11.
13. Young TJ, Jubery TZ, Carley CN, Carroll M, Sarkar S, Singh AK, et al. "Canopy fingerprints" for characterizing three-dimensional point cloud data of soybean canopies. Frontiers in Plant Science 2023;14:1141153.
14. Li Z, Guo R, Li M, Chen Y, Li G. A review of computer vision technologies for plant phenotyping. Computers and Electronics in Agriculture 2020;176:105672.
15. CloudCompare, CloudCompare (Version 2.11.1); 2020. https://github.com/CloudCompare/CloudCompare/releases/

tag/v2.11.1.

16. Nutter Jr F, Gleason M, Jenco J, Christians N. Assessing the accuracy, intra-rater repeatability, and inter-rater reliability of disease assessment systems. Phytopathology 1993;83(8):806–812.
17. Golbach F, Kootstra G, Damjanovic S, Otten G, van de Zedde R. Validation of plant part measurements using a 3D reconstruction method suitable for high-throughput seedling phenotyping. Machine Vision and Applications 2016;27:663–680.
18. Nguyen CV, Fripp J, Lovell DR, Furbank R, Kuffner P, Daily H, et al. 3D scanning system for automatic high-resolution plant phenotyping. In: 2016 international conference on digital image computing: techniques and applications (DICTA) IEEE; 2016. p. 1–8.
19. Community Plant Varienty Office. Protocol for tests on distinctness, uniformity and stability: Sugarbeet Components. Community Plant Varienty Office; 2018.
20. Hosoi F, Nakai Y, Omasa K. Estimating the leaf inclination angle distribution of the wheat canopy using a portable scanning lidar. Journal of Agricultural Meteorology 2009;65:297–302. https://api.semanticscholar.org/CorpusID:73530816.
21. Xiang L, Gai J, Bao Y, Yu J, Schnable PS, Tang L. Field-based robotic leaf angle detection and characterization of maize plants using stereo vision and deep convolutional neural networks. Journal of Field Robotics 2023;40:1034–1053.
22. Itakura K, Hosoi F. Automatic leaf segmentation for estimating leaf area and leaf inclination angle in 3D plant images. Sensors 2018;18(10):3576.
23. Biskup B, Scharr H, Schurr U, Rascher U. A stereo imaging system for measuring structural parameters of plant canopies. Plant, cell & environment 2007;30(10):1299–1308.
24. Dandrifosse S, Bouvry A, Leemans V, Dumont B, Mercatoris B. Imaging wheat canopy through stereo vision: Overcoming the challenges of the laboratory to field transition for morphological features extraction. Frontiers in Plant Science 2020;11:96.
25. Müller-Linow M, Pinto-Espinosa F, Scharr H, Rascher U. The leaf angle distribution of natural plant populations: assessing the canopy with a novel software tool. Plant methods 2015;11:1–16.
26. Topp CN, Iyer-Pascuzzi AS, Anderson JT, Lee CR, Zurek PR, Symonova O, et al. 3D phenotyping and quantitative trait locus mapping identify core regions of the rice genome controlling root architecture. Proceedings of the National Academy of Sciences 2013;110(18):E1695–E1704.
27. Hanon MM, Zsidai L, Ma Q. Accuracy investigation of 3D printed PLA with various process parameters and different colors. Materials Today: Proceedings 2021;42:3089–3096.
28. Harris BD, Nilsson S, Poole CM. A feasibility study for using ABS plastic and a low-cost 3D printer for patient-specific brachytherapy mould design. Australasian physical & engineering sciences in medicine 2015;38:399–412.
29. Zhou QY, Park J, Koltun V, Open3D: A Modern Library for 3D Data Processing; 2020. https://www.open3d.org/.
30. Bernardini F, Mittleman J, Rushmeier H, Silva C, Taubin G. The ball-pivoting algorithm for surface reconstruction. IEEE Transactions on Visualization and Computer Graphics 1999;5(4):349–359.
31. Rusu RB, Cousins S. 3D is here: Point Cloud Library (PCL). In: IEEE International Conference on Robotics and Automation (ICRA) Shanghai, China; 2011. .
32. Blender Online Community. Blender - a 3D modelling and rendering package. Blender Foundation, Stichting Blender Foundation, Amsterdam; 2018, http://www.blender.org.
33. Santana L, Alves JL, Sabino Netto AdC, Merlini C. A comparative study between PETG and PLA for 3D Printing through thermal, chemical and mechanical characterization. Matéria (Rio de Janeiro) 2018;23.
34. Srinivasan R, Prathap P, Raj A, Kannan SA, Deepak V. Influence of fused deposition modeling process parameters on the mechanical properties of PETG parts. Materials Today: Proceedings 2020;27:1877–1883.
35. Prusa3D, PrusaSlicer (Version 2.4.0-beta1); 2021. https://github.com/prusa3d/PrusaSlicer/releases/tag/version_2.4.0-beta1.
36. Silva C, Pais AI, Caldas G, Gouveia BP, Alves JL, Belinha J. Study on 3D printing of gyroid-based structures for superior structural behaviour. Progress in Additive Manufacturing 2021;6(4):689–703.
37. Hanon MM, Marczis R, Zsidai L. Anisotropy evaluation of different raster directions, spatial orientations, and fill percentage of 3D printed PETG tensile test specimens. Key engineering materials 2019;821:167–173.
38. Sepahi MT, Abusalma H, Jovanovic V, Eisazadeh H. Mechanical properties of 3D-printed parts made of polyethylene terephthalate glycol. Journal of Materials Engineering and Performance 2021;30:6851–6861.
39. Nieto DM, López VC, Molina SI. Large-format polymeric pellet-based additive manufacturing for the naval industry. Additive Manufacturing 2018;23:79–85.
40. Paulus S, Behmann J, Mahlein AK, Plümer L, Kuhlmann H. Low-cost 3D systems: suitable tools for plant phenotyping. Sensors 2014;14(2):3001–3018.
41. Magistri F, Chebrolu N, Stachniss C. Segmentation-based 4D registration of plants point clouds for phenotyping. In: 2020 IEEE/RSJ International Conference on Intelligent Robots and Systems (IROS); 2020. p. 2433–2439.
42. Paulus S, Dupuis J, Mahlein AK, Kuhlmann H. Surface feature based classification of plant organs from 3D laserscanned point clouds for plant phenotyping. BMC bioinformatics 2013;14(1):1–12.
43. Paulus S, Dupuis J, Riedel S, Kuhlmann H. Automated analysis of barley organs using 3D laser scanning: An approach for high throughput phenotyping. Sensors 2014;14(7):12670–12686.
44. Rose JC, Paulus S, Kuhlmann H. Accuracy analysis of a multi-view stereo approach for phenotyping of tomato plants at the organ level. Sensors 2015;15(5):9651–9665.
45. Chen Y, Medioni G. Object modelling by registration of multiple range images. Image and vision computing 1992;10(3):145–155.
46. Lague D, Brodu N, Leroux J. Accurate 3D comparison of complex topography with terrestrial laser scanner: Application to the Rangitikei canyon (NZ). ISPRS journal of photogrammetry and remote sensing 2013;82:10–26.
47. py4dgeo Development Core Team, py4dgeo: library for change analysis in 4D point clouds (Version 0.5.0); 2022. https://github.com/3dgeo-heidelberg/py4dgeo.
48. Van Rossum G, Drake FL. Python 3 Reference Manual. Scotts Valley, CA: CreateSpace; 2009.
49. Harris CR, Millman KJ, van der Walt SJ, Gommers R, Virtanen P, Cournapeau D, et al. Array programming with NumPy. Nature 2020 Sep;585(7825):357–362. https://doi.org/10.1038/s41586-020-2649-2.
50. Sharp N, Potpourri3D (Version 0.0.8); 2021. https://github.com/nmwsharp/potpourri3d/releases/tag/v0.0.8.
51. Bellock K, Alphashape (Version 1.3.1); 2021. https://github.com/bellockk/alphashape/releases/tag/v1.3.1.
52. Gillies S, Descartes (Version 1.1.0); 2017. https://pypi.org/project/descartes/#description.
53. Barber CB, Dobkin DP, Huhdanpaa H. The quickhull algorithm for convex hulls. ACM Transactions on Mathematical Software (TOMS) 1996;22(4):469–483. http://www.qhull.org.
54. Crane K, Weischedel C, Wardetzky M. The Heat Method for Distance Computation. Commun ACM 2017 Oct;60(11):90–99. http://doi.acm.org/10.1145/3131280.
55. Esser F, Rosu RA, Corneließen A, Klingbeil L, Kuhlmann H, Behnke S. Field Robot for High-Throughput and High-

Resolution 3D Plant Phenotyping: Towards Efficient and Sustainable Crop Production. IEEE Robotics & Automation Magazine 2023;30:20–29.
56. Rosu RA, Behnke S. PermutoSDF: Fast Multi-View Reconstruction with Implicit Surfaces Using Permutohedral Lattices. In: 2023 IEEE/CVF Conference on Computer Vision and Pattern Recognition (CVPR); 2023. p. 8466–8475.
57. Medic T, Bömer J, Paulus S. Challenges and Recommendations for 3d Plant Phenotyping in Agriculture Using Terrestrial Lasers Scanners. ISPRS Annals of the Photogrammetry, Remote Sensing and Spatial Information Sciences 2023 Dec;X-1/W1-2023:1007–1014. http://dx.doi.org/10.5194/isprs-annals-X-1-W1-2023-1007-2023.
58. Griffiths M. A 3D print repository for plant phenomics. Plant Phenomics 2020;.
59. Liang T, Knappett J, Bengough A, Ke Y. Small-scale modelling of plant root systems using 3D printing, with applications to investigate the role of vegetation on earthquake-induced landslides. Landslides 2017;14:1747–1765.
60. Romeijn T, Singh K, Behrens M, Paul G. Effect of accelerated weathering on the creep behaviour of additively manufactured Polyethylene Terephthalate Glycol (PETG). Journal of Polymer Research 2021;28(9):352.
61. Yuen PK. Embedding objects during 3D printing to add new functionalities. Biomicrofluidics 2016;10(4).
62. Kannan S, Ramamoorthy M, Sudhagar E, Gunji B. Mechanical characterization and vibrational analysis of 3D printed PETG and PETG reinforced with short carbon fiber. In: AIP Conference Proceedings, vol. 2270; 2020. p. 030004.
63. Holcomb G, Caldona EB, Cheng X, Advincula RC. On the optimized 3D printing and post-processing of PETG materials. MRS communications 2022;12(3):381–387.
64. Magistri F, Marks E, Nagulavancha S, Vizzo I, Läebe T, Behley J, et al. Contrastive 3D Shape Completion and Reconstruction for Agricultural Robots Using RGB-D Frames. IEEE Robotics and Automation Letters 2022;7(4):10120–10127.
65. Pan Y, Magistri F, Läbe T, Marks E, Smitt C, McCool C, et al. Panoptic Mapping with Fruit Completion and Pose Estimation for Horticultural Robots. In: 2023 IEEE/RSJ International Conference on Intelligent Robots and Systems (IROS); 2023. p. 4226–4233.

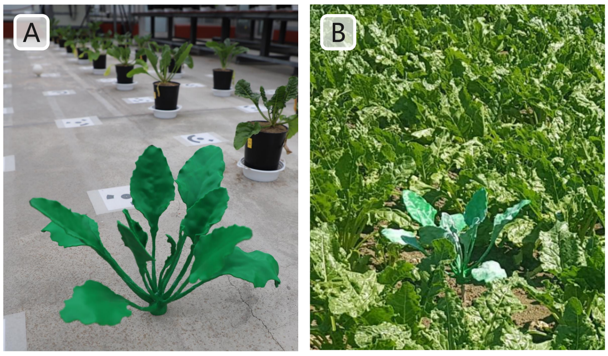

Figure 1. The proposed 3D printed reference model for sugar beet in A) greenhouse and B) field experiments.

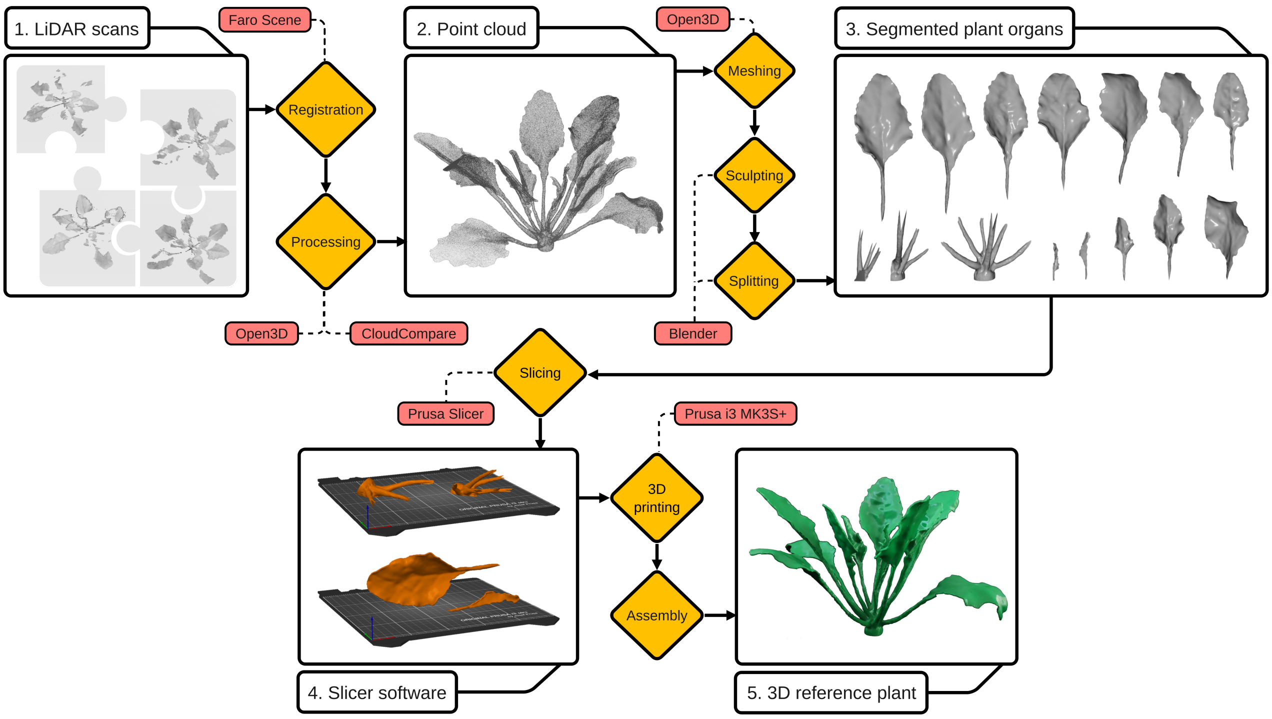

Figure 2. Workflow for the creation of the artificial sugar beet reference plant. The intermediate processes are shown in white boxes. Conducted production steps are highlighted in orange while the used soft- and hardware is pictured in light red.

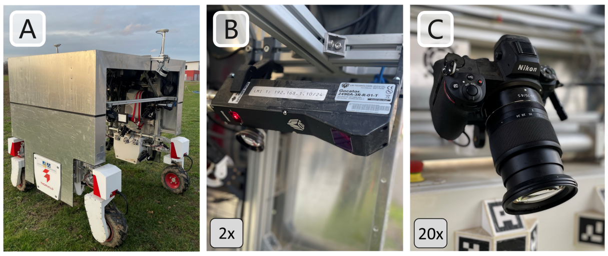

Figure 3. A) Field robot phenotyping platform equipped with B) two laser line scanners (LMI Gocator 2490) and C) twenty DSLM-cameras (Nikon Z7). More details about the platform are provided by Esser et al. [55].

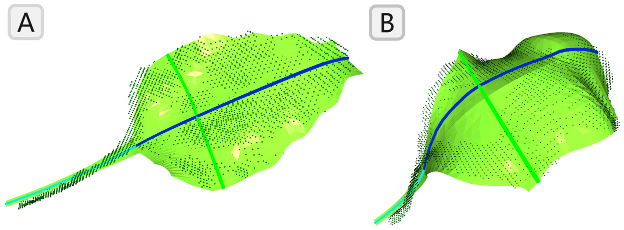

Figure 4. Display of leaf models fitted to the point cloud of the reference model for A) leaf 7 and B) leaf 1. The marked lines indicate the sections used to measure leaf length, blade length and blade width. More details about the algorithm are provided by Marks et al. [11].

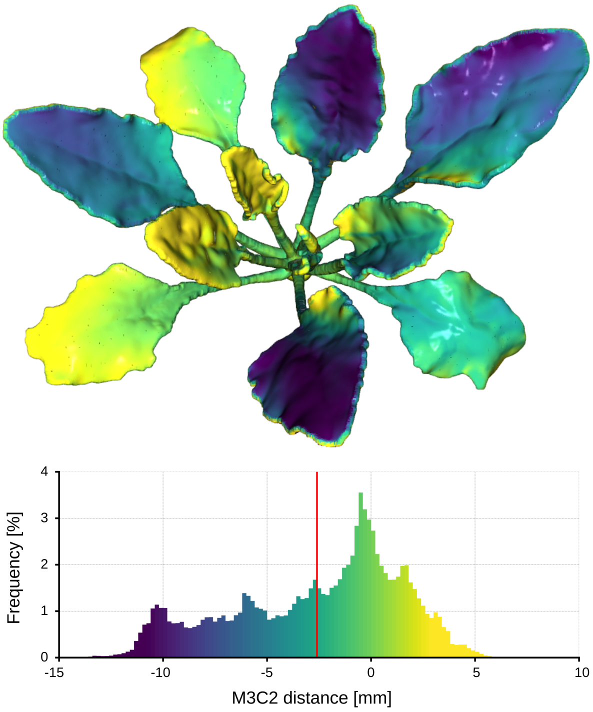

Figure 5. Differences of the computer model and the 3D printed reference model immediately after production, depicted by the M3C2 distance.

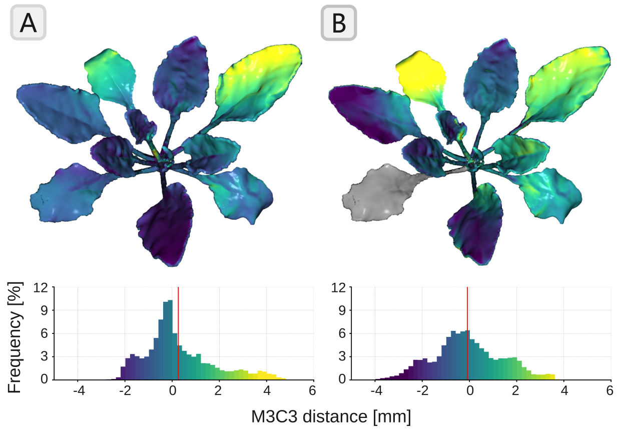

Figure 6. Dimensional stability of the 3D printed reference model demonstrated by the M3C2 distance between the model immediately after production and the model after A) 143 days and B) 361 days of intensive use. In segment B), one leaf is highlighted in grey, which was not included in the analysis because it was damaged by improper handling.

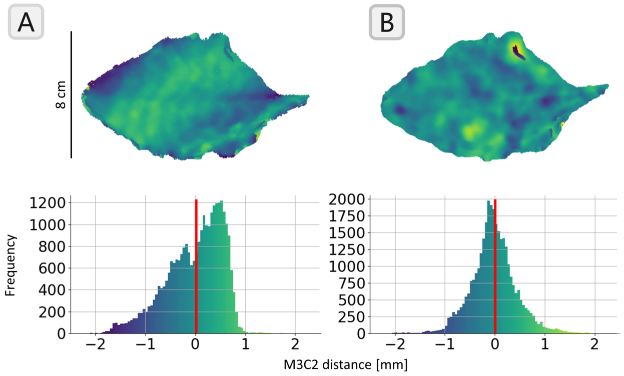

Figure 7. M3C2 distances w.r.t the reference scan for A) the laser line scanner system and B) the DSLM-camera system of the robotic phenotyping platform.

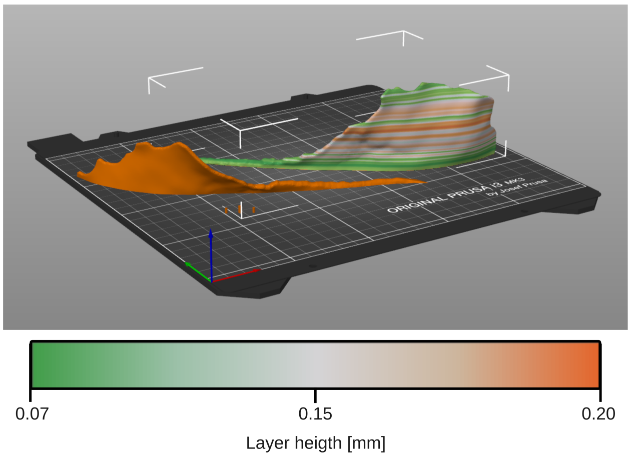

Figure 8. Improved printing orientation and variable layer height illustration (colored scale) for individual leaves. The leaf is cut along its longitudinal axes and assembled after printing.
