# Supplementary material for: A 3D printed plant model for accurate and reliable 3D plant phenotyping
Source: Gigascience. 2024 Jun 20;13:giae035. doi: 10.1093/gigascience/giae035 (PMC11186670; doi:10.1093/gigascience/giae035)
Supplement: giae035_Response_to_Reviewer_Comments_Revision_1 [file giae035_response_to_reviewer_comments_revision_1.pdf]

Author's Response To Reviewer Comments

Dear Ms. Nogoy,

I am sending you my updated manuscript. The changes you mentioned have been made as follows:

- RRIDs have been added for Python and Numpy.
- The data availability section has been edited according to your comments and the dataset has been cited accordingly. In addition, based on your email of 5/21/2024, the section has been adjusted and links to Thingiverse and Sketchfab have been added.

I hope that everything has been edited to your satisfaction. If there are any changes you would like made, please let me know.

Thanks for your professional work.

Sincerely yours,
Jonas Bömer
