# Supplementary material for: A 3D printed plant model for accurate and reliable 3D plant phenotyping
Source: Gigascience. 2024 Jun 20;13:giae035. doi: 10.1093/gigascience/giae035 (PMC11186670; doi:10.1093/gigascience/giae035)
Supplement: giae035_Response_to_Reviewer_Comments_Original_Submission [file giae035_response_to_reviewer_comments_original_submission.pdf]

Author's Response To Reviewer Comments

Dear Ms. Nogoy,

I am writing to express my gratitude for the feedback of the reviewers on our manuscript "A 3D printed plant model for accurate sensor reconstruction and reliable 3D plant phenotyping". The thorough review and constructive criticism have improved the quality and clarity of our research. I would like to thank you for the objective evaluation of our work.
We appreciate the time and effort you took to provide detailed suggestions for improvement. The recommendations have been carefully considered, and I am pleased to inform you that we have incorporated them into the revised version of the manuscript.
Enclosed with this letter, you will find a detailed response outlining the changes made in response to your feedback. We are confident that these changes have strengthened the overall quality and impact of our work.
Thank you again for your time, expertise, and dedication.

Sincerely,
Jonas Bömer

@ Shangpeng Sun

1. Please clarify the innovative points of this study. A precise printed 3D plant model is useful for 3D plant phenotyping. However, in this study, the 3D printing was implemented using a consumer-grade 3D printing technique, and the methods for 3D plant phenotypic traits extraction were commonly used in previous studies. The reviewer did not see enough innovative points/methods implemented in the study.

The innovative points of the study or the main contribution are addressed in line 102-112 in the .tex file. We've conducted changes to this section to make the innovative points easier to understand. As stated in the introduction of the manuscript, the main contribution of this paper is an exploration into the potential of utilizing a plant model that can be 3D printed with low effort by anyone as a precise referencing tool in 3D plant phenotyping. The innovation is the combination of these two topics and the discovered benefits for 3D plant phenotyping. Besides the print files of our model, we provide a detailed guide for other researchers to follow and explore the possibilities of creating new reference models of various crops including complete plants or single plant organs. We have identified and evaluated three different use cases for a 3D printed reference model. One of the greatest possibilities is possibly in the opportunity to standardize evaluation of 3D sensors and algorithms in 3D plant phenotyping using 3D printing.

2. Compared to using an ordinary fake plant, what are the advantages for the 3D printing technique? If we have the phenotypic traits of the fake plant via computer vision-based methods or manual measurements, the plant also can be used for the phenotyping applications mentioned in this study. Please clarify.

Thank you for pointing this out. There are several advantages of our proposed method compared to using

an ordinary fake plant. The most important one is the possibility of 3D printable reference models to standardise reference approaches in 3D plant phenotyping since the models can be produced with high accuracy and low effort by the researchers themselves.

We've added this and other arguments in the section "3D printing for referencing in 3D plant phenotyping" of the discussion (line 356 in the .tex file).

3. The title 'A 3D printed plant model for accurate sensor reconstruction…', not fully understand the expression 'for accurate sensor reconstruction'.

This expression refers to the first use case presented in the paper, comparing different reconstructions from different sensors. But we see your point and discussed changing the title to "A 3D printed plant model for accurate and reliable 3D plant phenotyping". Change are made to line 31 in the .tex file.

4. What are the limitations of the 3D printing methods?

This is an important point that was only casually discussed in the paper. In addition to the broadly described advantages of using 3D printing in 3D plant phenotyping, there are also possible limitations to consider. To give a more general view on the topic, we've added a paragraph dedicated to the limitations of 3D printing methods in plant phenotyping. To not loose the focus of the evaluation of our own reference plant, we added it to the section of "Future Scenarios" in the discussion (line 424 in the .tex file).

@Michael Pound

1. As I mention above, this work is only applied to a single species. To what extent are the edits and considerations for printing that you have applied to sugar beet here applicable to other species, e.g. thinner leaves and stems like wheat? Do the conclusions your draw in the paper also apply to other species? I am not a crop scientist, but instinctively I feel that sugar beet has larger / more stable leaves and stems than some other species. Even if you are not planning to include other species, it would be worth a discussion of how general you believe this solution will be. I think as it stands this is the primary weakness of the paper that could be better addressed.

This point was already part of the discussion in section "Future Scenarios", but was only discussed shortly. Following your thoughts, we've dived a little deeper into the applicable of our work to other species and added some of our ideas on how to transfer our findings and conclutions. Changes are made in line 420-422 of the .tex file.

2. During 3D printing, what infilling settings did you use inside? Was the model 100% filled, or some lower %? Would this have an effect on the durability?

The print settings including infill settings are already described in the "Material and Methods" section in the subsection "3D printing" (line 126 in the .tex file). The effect of infill density and different infill patterns are discussed in the discussion in subsection "3D printing for referencing in 3D plant phenotyping" (line 374 in the .tex file).

3. In your section on morphological benchmark parameters, a figure might help here to visualise the descriptions. Your descriptions are good, but a diagram will make this easier to follow.

We agree that this section should be followed with focus. However, it is crucial to describe the benchmark parameters clearly and precisely, as they are measured from a specific starting point to an endpoint. This information serves as the basis for comparing sensors or algorithms with our benchmark parameters and cannot be accurately expressed in a graphical way. To prevent any misunderstandings, we have chosen to present the characteristics in text form.

4. I felt that table 2 was somewhat of a deluge of data, which makes it hard to compare. I am not sure what an alternative is, but scanning across looking for comparable values between columns is a challenge. Perhaps a series of small scatter plots? Or a small number simply to help illustrate the points being made? Alternatively you could show error as a percentage or some other metric.

Table 2 (and Table 1) provides benchmark data for some of the morphology features of the reference models; the intent is to provide a reference for other researchers, not to facilitate a visual comparison between automated and manual measurements. We are happy to share the print files and benchmark values to enable other researchers to use our model for their work. Because of this, it's important to present the raw values in a tabular form rather than in graphical representations. We decided against including a percentage error rate (as shown in Table 3-5) as it would only add confusion to the table. With that in mind, we've decided to keep the table in its current form.

5. Later in the discussion you talk about the laser and camera systems failing to reconstruct some inner leaves - can this be visualised? The argument of using this reference model to detect weaknesses in algorithms is good, but as with some of my other comments, a figure here might help better explain the point you are making.

We tried to visualize the two innermost leaves, but could not come up with a satisfactory 2D representation of the point clouds that would be useful to the reader, e.g., to identify a specific failure pattern of a sensor. In order to keep the number of figures in the article to a reasonable level, we decided to keep only the graphics that were most relevant to the article.
