# Supplementary material for: A 3D printed plant model for accurate and reliable 3D plant phenotyping
Source: Gigascience. 2024 Jun 20;13:giae035. doi: 10.1093/gigascience/giae035 (PMC11186670; doi:10.1093/gigascience/giae035)
Supplement: giae035_Reviewer_1_Report_Original_Submission — Chris Armit -- 2/7/2024 [file giae035_reviewer_1_report_original_submission.pdf]

Reviewer Report

Title: A 3D printed plant model for accurate and reliable 3D plant phenotyping

Version: Original Submission **Date:** 2/7/2024

Reviewer name: Chris Armit

Reviewer Comments to Author:

This excellent Research Article explores the highly novel concept of using a 3D printed model of a sugar beet plant as a reference spatial framework for plant phenotyping. The authors highlight the challenges of 3D registration, and address these challenges in the context of their LiDAR-derived 3D model using a combination of spatial deformation and deviation in morphological parameters. The colourmaps in the Figures are particularly illustrative, and are an effective means of visualising the difference between, for example, the 3D printed model and a conventional statistical computer model. In support of the manuscript, the LiDAR Scans, 3D Reconstructions, and Spatial Data have been submitted to the GigaScience DataBase (GigaDB) and include the following:
* Raw LiDAR scans of the greenhouse sugar beet plant used for generating the reference model
* Global registered LiDAR scans of the greenhouse sugar beet plant used for generating the reference model
* Digital point cloud of the reference plant model
* Registered laser scans used for analysing production deviations and deformations over time (dimensional stability)
* Reconstructions of the laser phenotyping system and the camera phenotyping system including a scan of the reference model (use case 1)
* Point cloud of single leaves used to extract benchmark parameters and evaluate extraction algorithms (use case 2)
* LiDAR point clouds used for extracting morphological parameters (use case 3)
* Print files (STL format)

I can confirm that the point cloud files can be visualised using CloudCompare, and that the 3D surface reconstructions can be visualised using MeshLab.
I thank the authors for submitting a very interesting manuscript that highlights the challenges and utility of 3D spatial mapping.

Methods

Are the methods appropriate to the aims of the study, are they well described, and are necessary controls included? Choose an item.

Conclusions

Are the conclusions adequately supported by the data shown? Choose an item.

Reporting Standards

Does the manuscript adhere to the journal's guidelines on minimum standards of reporting? Choose an item.

Choose an item.

Statistics

Are you able to assess all statistics in the manuscript, including the appropriateness of statistical tests used? Choose an item.

Quality of Written English

Please indicate the quality of language in the manuscript: Choose an item.

Declaration of Competing Interests

Please complete a declaration of competing interests, considering the following questions:

- Have you in the past five years received reimbursements, fees, funding, or salary from an organisation that may in any way gain or lose financially from the publication of this manuscript, either now or in the future?
- Do you hold any stocks or shares in an organisation that may in any way gain or lose financially from the publication of this manuscript, either now or in the future?
- Do you hold or are you currently applying for any patents relating to the content of the manuscript?
- Have you received reimbursements, fees, funding, or salary from an organization that holds or has applied for patents relating to the content of the manuscript?
- Do you have any other financial competing interests?
- Do you have any non-financial competing interests in relation to this paper?

If you can answer no to all of the above, write 'I declare that I have no competing interests' below. If your reply is yes to any, please give details below.

I work for GigaScience where I perform checks on the quality of image data and the image analysis approach. My funding is not dependent on the outcome of this review, and I declare that I have no competing interests.

I agree to the open peer review policy of the journal. I understand that my name will be included on my report to the authors and, if the manuscript is accepted for publication, my named report including any attachments I upload will be posted on the website along with the authors' responses. I agree for my report to be made available under an Open Access Creative Commons CC-BY license (http://creativecommons.org/licenses/by/4.0/). I understand that any comments which I do not wish to

be included in my named report can be included as confidential comments to the editors, which will not be published.

Choose an item.

To further support our reviewers, we have joined with Publons, where you can gain additional credit to further highlight your hard work (see: https://publons.com/journal/530/gigascience). On publication of this paper, your review will be automatically added to Publons, you can then choose whether or not to claim your Publons credit. I understand this statement.

Yes Choose an item.
