# Supplementary material for: A 3D printed plant model for accurate and reliable 3D plant phenotyping
Source: Gigascience. 2024 Jun 20;13:giae035. doi: 10.1093/gigascience/giae035 (PMC11186670; doi:10.1093/gigascience/giae035)
Supplement: giae035_Reviewer_1_Report_Revision_1 — Chris Armit -- 4/17/2024 [file giae035_reviewer_1_report_revision_1.pdf]

Reviewer Report

Title: A 3D printed plant model for accurate and reliable 3D plant phenotyping

Version: Revision 1 **Date:** 4/17/2024

Reviewer name: Chris Armit

Reviewer Comments to Author:

I have been invited to re-review this Research Article, which explores the highly novel concept of using a 3D printed model of a sugar beet plant as a reference spatial framework for plant phenotyping. As stipulated in my initial review, the authors highlight the challenges of 3D registration, and address these challenges in the context of their LiDAR-derived 3D model using a combination of spatial deformation and deviation in morphological parameters. I can confirm that the point cloud files can be visualised using CloudCompare, and that the 3D surface reconstructions can be visualised using MeshLab.

In addition, I confirm that the authors have sufficiently addressed the queries of Reviewers 2 and 3. In particular, the authors have detailed the innovative points of this study - namely that of utilising a 3D printed plant model as a precise referencing tool in 3D plant phenotyping - which was one of the salient remarks from Reviewer 2. Furthermore, in the updated "Future Scenarios" section the authors have additionally highlighted how the 3D phenotyping approach outlined in this Research Article can be applied to other plant species, such as wheat or maize, which was one of the major comments from Reviewer 3.

Of note, Reviewer 3 made the comment that: "table 2 was somewhat of a deluge of data, which makes it hard to compare". Table 2 includes the morphological parameters of the 3D reference model at leaf scale, and I personally find it helpful to include these details in the manuscript. In response to this comment, the authors state in their response to Reviewer 3 that: "Table 2 (and Table 1) provides benchmark data for some of the morphology features of the reference models; the intent is to provide a reference for other researchers, not to facilitate a visual comparison between automated and manual measurements" and for this reason the authors have "decided to keep the table in its current form". For a benchmarking study that aims to report on morphological benchmark parameters, I think this is a perfectly reasonable response.

In addition, Reviewer 3 invited the authors to provide a figure / diagram to help visualise the descriptions of morphological benchmark parameters. Importantly, Reviewer 3 states: "Your descriptions are good, but a diagram will make this easier to follow". In response, the authors state that: "This information serves as the basis for comparing sensors or algorithms with our benchmark parameters and cannot be accurately expressed in a graphical way. To prevent any misunderstandings, we have chosen to present the characteristics in text form." Whereas I see value in a Graphical Abstract / Figure as suggested by Reviewer 3, I understand the importance of the detailed descriptions of morphological benchmark parameters as provided by the authors. Importantly, the authors additionally state: "We are happy to share the print files and benchmark values to enable other researchers to use our model for their work. Because of this, it's important to present the raw values in a tabular form rather than in graphical representations". I think this is a reasonable response to Reviewer 3.

I thank the authors for submitting a very interesting manuscript that highlights the challenges and utility of 3D spatial mapping in the context of plant phenotyping.

Methods

Are the methods appropriate to the aims of the study, are they well described, and are necessary controls included? Choose an item.

Conclusions

Are the conclusions adequately supported by the data shown? Choose an item.

Reporting Standards

Does the manuscript adhere to the journal's guidelines on minimum standards of reporting? Choose an item.

Choose an item.

Statistics

Are you able to assess all statistics in the manuscript, including the appropriateness of statistical tests used? Choose an item.

Quality of Written English

Please indicate the quality of language in the manuscript: Choose an item.

Declaration of Competing Interests

Please complete a declaration of competing interests, considering the following questions:

- Have you in the past five years received reimbursements, fees, funding, or salary from an organisation that may in any way gain or lose financially from the publication of this manuscript, either now or in the future?
- Do you hold any stocks or shares in an organisation that may in any way gain or lose financially from the publication of this manuscript, either now or in the future?
- Do you hold or are you currently applying for any patents relating to the content of the manuscript?
- Have you received reimbursements, fees, funding, or salary from an organization that holds or has applied for patents relating to the content of the manuscript?
- Do you have any other financial competing interests?
- Do you have any non-financial competing interests in relation to this paper?

If you can answer no to all of the above, write 'I declare that I have no competing interests' below. If your reply is yes to any, please give details below.

I work for GigaScience where I perform checks on the quality of image data and the image analysis approach. My funding is not dependent on the outcome of this review, and I declare that I have no competing interests.

I agree to the open peer review policy of the journal. I understand that my name will be included on my report to the authors and, if the manuscript is accepted for publication, my named report including any attachments I upload will be posted on the website along with the authors' responses. I agree for my report to be made available under an Open Access Creative Commons CC-BY license (http://creativecommons.org/licenses/by/4.0/). I understand that any comments which I do not wish to be included in my named report can be included as confidential comments to the editors, which will not be published.

Choose an item.

To further support our reviewers, we have joined with Publons, where you can gain additional credit to further highlight your hard work (see: https://publons.com/journal/530/gigascience). On publication of this paper, your review will be automatically added to Publons, you can then choose whether or not to claim your Publons credit. I understand this statement.

Yes Choose an item.
