# Supplementary material for: A 3D printed plant model for accurate and reliable 3D plant phenotyping
Source: Gigascience. 2024 Jun 20;13:giae035. doi: 10.1093/gigascience/giae035 (PMC11186670; doi:10.1093/gigascience/giae035)
Supplement: giae035_Reviewer_2_Report_Original_Submission — Shangpeng Sun -- 2/26/2024 [file giae035_reviewer_2_report_original_submission.pdf]

Reviewer Report

Title: A 3D printed plant model for accurate and reliable 3D plant phenotyping

Version: Original Submission **Date:** 2/26/2024

Reviewer name: Shangpeng Sun

Reviewer Comments to Author:

This manuscript applied a consumer-grade 3D printing technique FDM to produce 3D plant models. Then, three use cases were proposed for the application of the reference model: evaluating and comparing 3D sensor systems, investigating the potential accuracy of parameter extraction algorithms, and continuously monitoring these algorithms in practical experiments in greenhouse and field experiments. A precise and accurate 3D plant model is useful for plant phenotyping applications. The comments are described as follows:

1. Please clarify the innovative points of this study. A precise printed 3D plant model is useful for 3D plant phenotyping. However, in this study, the 3D printing was implemented using a consumer-grade 3D printing technique, and the methods for 3D plant phenotypic traits extraction were commonly used in previous studies. The reviewer did not see enough innovative points/methods implemented in the study.
2. Compared to using an ordinary fake plant, what are the advantages for the 3D printing technique? If we have the phenotypic traits of the fake plant via computer vision-based methods or manual measurements, the plant also can be used for the phenotyping applications mentioned in this study. Please clarify.
3. The title 'A 3D printed plant model for accurate sensor reconstruction…', not fully understand the expression 'for accurate sensor reconstruction'.
4. What are the limitations of the 3D printing methods?

Methods

Are the methods appropriate to the aims of the study, are they well described, and are necessary controls included? Choose an item.

Conclusions

Are the conclusions adequately supported by the data shown? Choose an item.

Reporting Standards

Does the manuscript adhere to the journal’s guidelines on [minimum standards of reporting?]() Choose an item.

Choose an item.

Statistics

Are you able to assess all statistics in the manuscript, including the appropriateness of statistical tests used? Choose an item.

Quality of Written English

Please indicate the quality of language in the manuscript: Choose an item.

Declaration of Competing Interests

Please complete a declaration of competing interests, considering the following questions:

- Have you in the past five years received reimbursements, fees, funding, or salary from an organisation that may in any way gain or lose financially from the publication of this manuscript, either now or in the future?
- Do you hold any stocks or shares in an organisation that may in any way gain or lose financially from the publication of this manuscript, either now or in the future?
- Do you hold or are you currently applying for any patents relating to the content of the manuscript?
- Have you received reimbursements, fees, funding, or salary from an organization that holds or has applied for patents relating to the content of the manuscript?
- Do you have any other financial competing interests?
- Do you have any non-financial competing interests in relation to this paper?

If you can answer no to all of the above, write 'I declare that I have no competing interests' below. If your reply is yes to any, please give details below.

I declare that I have no competing interests

I agree to the open peer review policy of the journal. I understand that my name will be included on my report to the authors and, if the manuscript is accepted for publication, my named report including any attachments I upload will be posted on the website along with the authors' responses. I agree for my report to be made available under an Open Access Creative Commons CC-BY license (http://creativecommons.org/licenses/by/4.0/). I understand that any comments which I do not wish to be included in my named report can be included as confidential comments to the editors, which will not be published.

Choose an item.

To further support our reviewers, we have joined with Publons, where you can gain additional credit to further highlight your hard work (see: https://publons.com/journal/530/gigascience). On publication of this paper, your review will be automatically added to Publons, you can then choose whether or not to claim your Publons credit. I understand this statement.

Yes Choose an item.
