# Supplementary material for: A 3D printed plant model for accurate and reliable 3D plant phenotyping
Source: Gigascience. 2024 Jun 20;13:giae035. doi: 10.1093/gigascience/giae035 (PMC11186670; doi:10.1093/gigascience/giae035)
Supplement: giae035_Reviewer_3_Report_Original_Submission — Michael Pound -- 3/30/2024 [file giae035_reviewer_3_report_original_submission.pdf]

Reviewer Report

Title: A 3D printed plant model for accurate and reliable 3D plant phenotyping

Version: Original Submission **Date:** 3/30/2024

Reviewer name: Michael Pound

Reviewer Comments to Author:

The authors present a study on the use of a 3D printed plant for long-term phenotyping applications. They aim to create a realistic and accurate 3D model of sugar beet, measure the accuracy of reconstruction, and then study the long term effect of the use of this model in field conditions.
The paper is well written, grammatically very good and easy to follow. The introduction and background are very good, and the authors have made good efforts to outline the potential benefits of 3D printing in this space. The main "drawback" of the paper, if there is one, is that this is a study on a single model of a single plant species. As a proof of concept this is reasonable, but of course the impact would be greater if we were also considering other (differently shaped) plants such as Wheat or Rice. Nevertheless, it seems that this is a viable solution for calibrating / testing 3D reconstruction algorithms in lab and field conditions, and over time. I have a few comments the authors might wish to address:

- As I mention above, this work is only applied to a single species. To what extent are the edits and considerations for printing that you have applied to sugar beet here applicable to other species, e.g. thinner leaves and stems like wheat? Do the conclusions your draw in the paper also apply to other species? I am not a crop scientist, but instinctively I feel that sugar beet has larger / more stable leaves and stems than some other species. Even if you are not planning to include other species, it would be worth a discussion of how general you believe this solution will be. I think as it stands this is the primary weakness of the paper that could be better addressed.
- During 3D printing, what infilling settings did you use inside? Was the model 100% filled, or some lower %? Would this have an effect on the durability?
- In your section on morphological benchmark parameters, a figure might help here to visualise the descriptions. Your descriptions are good, but a diagram will make this easier to follow.
- I felt that table 2 was somewhat of a deluge of data, which makes it hard to compare. I am not sure what an alternative is, but scanning across looking for comparable values between columns is a challenge. Perhaps a series of small scatter plots? Or a small number simply to help illustrate the points being made? Alternatively you could show error as a percentage or some other metric.
- Later in the discussion you talk about the laser and camera systems failing to reconstruct some inner leaves - can this be visualised? The argument of using this reference model to detect weaknesses in algorithms is good, but as with some of my other comments, a figure here might help better explain the point you are making.

Methods

Are the methods appropriate to the aims of the study, are they well described, and are necessary controls included? Choose an item.

Conclusions

Are the conclusions adequately supported by the data shown? Choose an item.

Reporting Standards

Does the manuscript adhere to the journal's guidelines on minimum standards of reporting? Choose an item.

Choose an item.

Statistics

Are you able to assess all statistics in the manuscript, including the appropriateness of statistical tests used? Choose an item.

Quality of Written English

Please indicate the quality of language in the manuscript: Choose an item.

Declaration of Competing Interests

Please complete a declaration of competing interests, considering the following questions:

- Have you in the past five years received reimbursements, fees, funding, or salary from an organisation that may in any way gain or lose financially from the publication of this manuscript, either now or in the future?
- Do you hold any stocks or shares in an organisation that may in any way gain or lose financially from the publication of this manuscript, either now or in the future?
- Do you hold or are you currently applying for any patents relating to the content of the manuscript?
- Have you received reimbursements, fees, funding, or salary from an organization that holds or has applied for patents relating to the content of the manuscript?
- Do you have any other financial competing interests?
- Do you have any non-financial competing interests in relation to this paper?

If you can answer no to all of the above, write 'I declare that I have no competing interests' below. If your reply is yes to any, please give details below.

I declare that I have no competing interests.

I agree to the open peer review policy of the journal. I understand that my name will be included on my report to the authors and, if the manuscript is accepted for publication, my named report including any attachments I upload will be posted on the website along with the authors' responses. I agree for my

report to be made available under an Open Access Creative Commons CC-BY license (http://creativecommons.org/licenses/by/4.0/). I understand that any comments which I do not wish to be included in my named report can be included as confidential comments to the editors, which will not be published.

Choose an item.

To further support our reviewers, we have joined with Publons, where you can gain additional credit to further highlight your hard work (see: https://publons.com/journal/530/gigascience). On publication of this paper, your review will be automatically added to Publons, you can then choose whether or not to claim your Publons credit. I understand this statement.

Yes Choose an item.
